# Photon and particle radiotherapy induce redundant modular chemotaxis of human lymphocytes

**DOI:** 10.1172/jci.insight.190149

**Published:** 2025-08-14

**Authors:** Joscha A. Kraske, Michael M. Allers, Aleksei Smirnov, Bénédicte Lenoir, Azaz Ahmed, Meggy Suarez-Carmona, Mareike Hampel, Damir Krunic, Alexandra Tietz-Dalfuß, Tizian Beikert, Jonathan M. Schneeweiss, Stephan Brons, Dorothee Albrecht, Thuy Trinh, Muzi Liu, Nathalia A. Giese, Christin Glowa, Jakob Liermann, Ramon Lopez Perez, Dirk Jäger, Jürgen Debus, Niels Halama, Peter E. Huber, Thomas Walle

**Affiliations:** 1 Department of Medical Oncology, National Center for Tumor Diseases (NCT), and; 2 Department of Radiation Oncology, Heidelberg University Hospital, Medical Faculty Heidelberg, Heidelberg University, Heidelberg, Germany.; 3 Division of Molecular and Radiooncology, German Cancer Research Center (DKFZ), Heidelberg, Germany.; 4 Clinical Cooperation Unit Applied Tumor Immunity and; 5 Division of Translational Immunotherapy, DKFZ, Heidelberg, Germany.; 6 Helmholtz Center for Translational Oncology (HI-TRON), Department of Cancer Immunology & Cancer Immunotherapy, Mainz, Germany.; 7 Light Microscopy Facility, DKFZ, Heidelberg, Germany.; 8 Heidelberg Ion Therapy Center (HIT), Department of Radiation Oncology, Heidelberg University Hospital, Heidelberg, Germany.; 9 Heidelberg Institute for Radiation Oncology (HIRO), Heidelberg, Germany.; 10 Department of General, Visceral and Transplantation Surgery, Heidelberg University Hospital, Medical Faculty Heidelberg, Heidelberg University, Germany.; 11 Division of Medical Physics in Radiation Oncology and; 12 Clinical Cooperation Unit Radiation Oncology, DKFZ, Heidelberg, Germany.; 13 Institute of Immunology, Heidelberg University Hospital, Medical Faculty Heidelberg, Heidelberg University, Heidelberg, Germany.

**Keywords:** Immunology, Oncology, Cellular senescence, Chemokines, Radiation therapy

## Abstract

Radiotherapy triggers chemokine release and leukocyte infiltration in preclinical models through activation of the senescence-associated secretory phenotype (SASP). However, effects of irradiation on senescence and SASP in human tissue and in the context of particle radiotherapy remain unclear. Here, we analyzed chemokine patterns after radiotherapy of human pancreatic tumors and cancer cell lines. We show that irradiated tumor cells coexpressed SASP chemokines in defined modules. These chemokine modules correlated with infiltration of distinct leukocyte subtypes expressing cognate receptors. We developed a patient-derived pancreatic tumor explant system, which verified our identified radiation-induced chemokine modules. Chemokine modules were partially conserved in cancer cells in response to photon and particle irradiation, showing a dose-dependent plateau effect, and induced subsequent migration of NK and T cell populations. Hence, our work reveals redundant interactions of cancer cells and immune cells in human tissue, suggesting that targeting multiple chemokines is required to efficiently perturb leukocyte infiltration after photon or particle radiotherapy.

## Introduction

Cellular senescence is a conserved stress response in which cells pause their regular cell cycle to mitigate damage (1–3). It is characterized by the permanent loss of proliferative capacity as well as distinct changes in cell morphology, metabolism, and function (1). Senescence does not only affect the cell itself, but it also influences bystander cells through the release of soluble mediators collectively referred to as the senescence-associated secretory phenotype (SASP) (4, 5).

Cytotoxic therapies, such as radiotherapy, initiate cellular senescence and SASP by generating DNA double-strand breaks (5, 6), which are detected by the DNA damage response machinery or the cytosolic cyclic GMP-AMP synthase (cGAS) (2).

The impact of radiation-induced cellular senescence on tumor growth is context dependent. SASP promotes tumor invasiveness and metastasis; however, SASP factors can also promote the infiltration of antitumor effector immune cells and improve immunotherapy outcomes (7–9).

The immune cell composition within the tumor microenvironment (TME) is controlled by chemokine signaling (10–12), which can be effectively altered by radiotherapy, leading to leukocyte recruitment (13). Given that the efficacy of radiotherapy partly relies on the induction of antitumor immunity through immune modulation and immunogenic cell death (13–15), combination treatment with immunotherapy is increasingly applied in cancer (16–18). Therefore, a deeper understanding of the molecular mechanisms underlying radiation-induced immune cell recruitment is important to optimize synergistic approaches with clinical relevance.

Individual chemokines have been described as part of the SASP after radiotherapy, leading to infiltration of immune cells, which, depending on the context, can either promote or suppress tumor growth (8, 19–22). However, it remains unclear whether radiotherapy-induced chemokine release is limited to context-dependent induction of individual chemokines, induces broad homogeneous chemokine upregulation, or induces a modular response pattern in which defined groups of chemokines are regulated uniformly. Along these lines, modularity of cytokine and cytokine receptor expression has been revealed as a critical determinant of pancreatic tumorigenesis (23). In humans, the composition of radiation-induced SASP is poorly understood, given the lack of available experimental model systems.

Conventional radiotherapy for solid tumors, including pancreatic adenocarcinoma (PDAC), usually uses small fractions of around 2 Gy photon radiation over 5–6 weeks to achieve cumulative doses of more than 50 Gy (24). However, radiotherapy is constantly adopting new technology, which may influence SASP patterns. Advances in imaging and radiation delivery techniques have led to an increase in the use of stereotactic body radiotherapy (SBRT), in which high fractions of radiation are delivered over a short period of time (e.g., 40–50 Gy cumulative dose over 4–5 days) (24–26). In addition, irradiation with charged particles, like protons and heavy ions, is increasingly adopted at cancer centers because of their advantageous physical characteristics allowing improved dose delivery to target volumes while sparing adjacent healthy tissue (27–30). Particle therapy has a higher relative biological effectiveness (RBE) in limiting clonogenic cell survival compared with photon therapy (27, 30, 31). However, its effects on SASP chemotactic patterns remain largely uncharacterized. Together, these advances in radiation oncology offer new opportunities to effectively target radioresistant tumors, such as PDAC, which have historically shown limited responsiveness to conventional radiotherapy.

Here, we systematically study the role of radiotherapy in chemokine release by human cancers. We reveal an unappreciated modularity of chemokine expression in irradiated human pancreatic tumors. Using a potentially novel explant system to study SASP in primary human pancreatic tumor samples (32–34), we experimentally confirmed expression of these chemokine modules after radiotherapy. Moreover, chemokines were potently induced in multiple cancer cell lines after photon and particle irradiation, showing a dose-dependent plateau effect and leading to T and NK cell chemotaxis. Collectively, our results suggest that combinatorial approaches targeting multiple chemokines simultaneously are necessary to effectively modify radiation-induced immune cell migration.

## Results

### Modular SASP chemokine expression and leukocyte infiltration in irradiated patients with PDAC.

**Figure 1:**
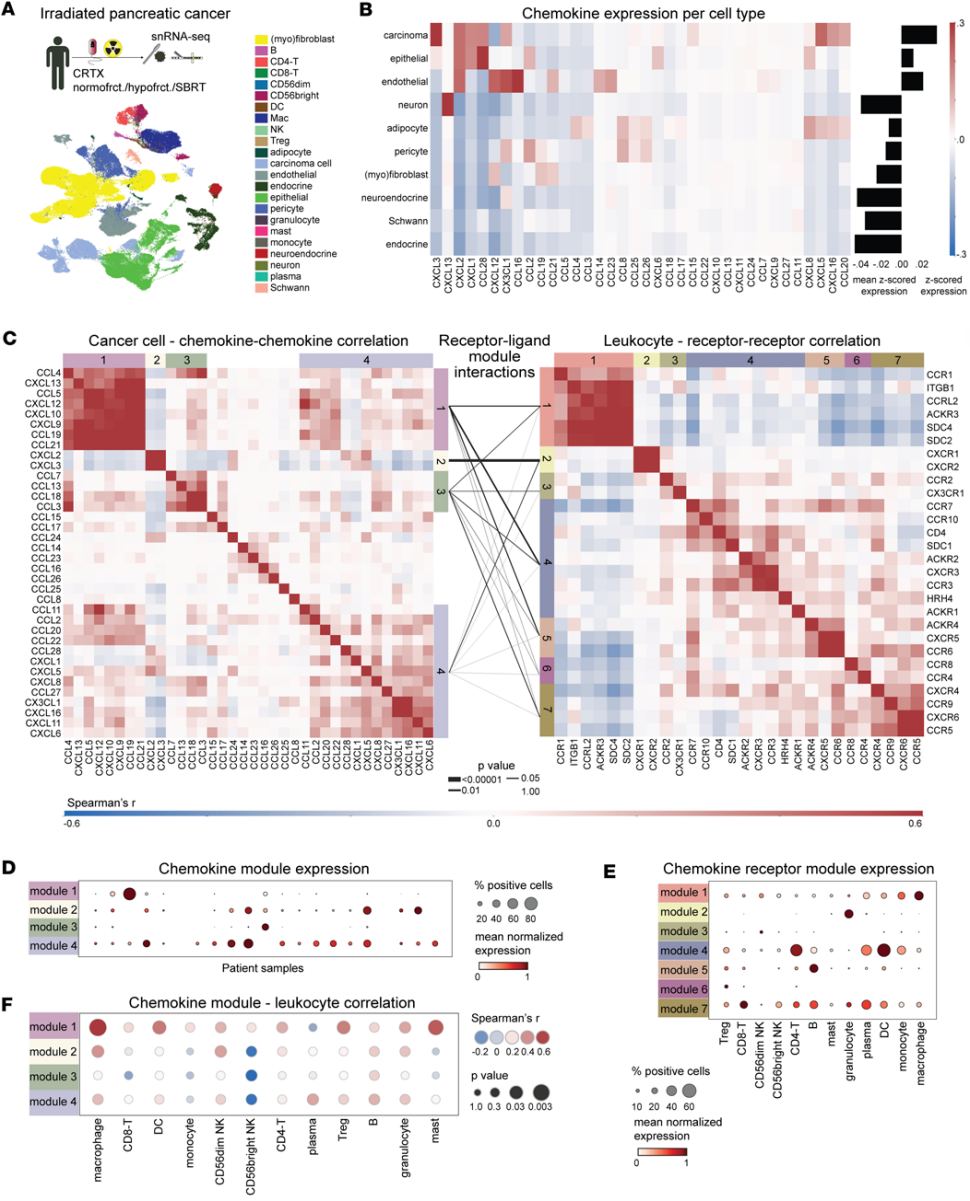
Modular chemokine expression enables redundant leukocyte cancer cell interactions in irradiated pancreatic cancer. (**A**) Schema and uniform manifold approximation and projection (UMAP) of single-nucleus RNA-sequencing data from pancreatic cancers after chemoradiotherapy from Hwang et al. (35) with color code indicating cell type (*n* = 97,987 cells). The most frequently applied protocols included FOLFIRINOX (± nivolumab, ± losartan) followed by hypofractionated (hypofrct.10 × 3 Gy), normofractionated (normofrct., 28 × 1.8 Gy), and stereotactic body radiotherapy (SBRT, 6 × 6 Gy) with concurrent capecitabine or 5-fluorouracil. (**B**) Chemokines in the irradiated pancreas are predominantly expressed in cancer cells. Heatmap indicating mean *z*-scored (across *n* = 97,987 cells) chemokine expression per cell type and row average per cell type. (**C**) Chemokine expression in cancer cells (left, *n* = 10,862) and chemokine receptor expression in immune cells (right, *n* = 9,768) is highly modular. Heatmaps indicating Spearman’s correlation coefficients and correlated chemokine/chemokine receptor modules retrieved by hierarchical clustering are highlighted by colored side bars. Connecting lines between modules indicate the significance of potential receptor–ligand interactions as estimated by a permutation test. (**D**) Chemokine modules are expressed across multiple patients (*n* = 21). (**E**) Chemokine receptor modules show cell type–specific expression (*n* = 12 cell types). (**F**) Chemokine modules are correlated with infiltration of specific leukocyte subsets expressing cognate receptors. Spearman’s correlation coefficients between mean chemokine module scores and abundance of infiltrating immune cells (as % of leukocytes) per patient (*n* = 21) are indicated as color code and the *P* values as dot sizes.

To identify cell type–specific patterns of chemokine expression, we analyzed a single-nucleus sequencing dataset of 21 patients with PDAC (97,987 cells), who received neoadjuvant photon chemoradiotherapy (normo-/hypofractionated or SBRT) (Figure 1A and Supplemental Figure 1, A and B; supplemental material available online with this article; https://doi.org/10.1172/jci.insight.190149DS1) (35). In the nonimmune cell populations, chemokines showed cell type–specific expression patterns, with cancer cells showing the highest chemokine expression after radiotherapy (Figure 1B). Cancer cells expressed multiple SASP-associated CC (e.g., CCL20) and CXC (e.g., CXCL1, CXCL8, CXCL16) chemokines, which have been shown to be induced by radiotherapy in preclinical models (20, 21, 36–38) (Figure 1B). Several chemokines were primarily expressed in cancer cells (CXCL16, CXCL5, CCL20, CXCL3) while other chemokines were preferentially expressed by endothelial cells (CXCL12) or fibroblasts (CCL19), suggesting that chemokine expression patterns in irradiated tumors may be greatly influenced by cell type composition (Figure 1B).

Cancer cells were the main nonimmune cell source of chemokines in the irradiated pancreatic cancer microenvironment (Figure 1B). We therefore asked whether these chemokines were modularly or homogenously expressed across cancer cells. Clustering of chemokine-chemokine correlation patterns revealed 4 distinct expression modules in cancer cells (Figure 1C), which were computationally robust and not driven by individual patients (Supplemental Figure 1C). While most patients expressed at least 1 module (19 of 21, 90.5%), we observed relevant interpatient heterogeneity (Figure 1D and Supplemental Figure 2). Module 4 included canonical SASP chemokines like CCL2, CXCL1, and CXCL8 (Figure 1C) and was expressed most promiscuously in 18 of 21 patients (85.7%) (Figure 1D). Module 3 was expressed in only 9 of 21 patients (42.9%) (Figure 1D) and included chemokines such as CCL3 and CCL7 (Figure 1C). Module 1 featured chemokines more strongly expressed on stromal cells, such as CCL19, CCL21, and CXCL12, but also the SASP chemokines CCL5 and CXCL10 (Figure 1C). Module 1 was strongly expressed in cancer cells from 2 patients, likely reflecting pancreatic cancer with a more mesenchymal phenotype (Figure 1D).

On immune cells, cognate chemokine receptors showed similar modularity, which was also computationally robust and not driven by individual patients (Figure 1C and Supplemental Figure 1C). Many modules were cell type specific (Figure 1E), like receptor module 3 (CD56^dim^ NK cells) and receptor module 2 (granulocytes). Receptor modules 1, 4, and 7 were expressed across multiple T, NK, and myeloid cell populations. Hence, our results suggest modular expression of both chemokines in cancer cells as well as their cognate receptors in leukocytes.

We hypothesized that the observed expression modularity may result in redundant interactions between modules of chemokines in cancer cells and modules of receptors in immune cells, which explain radiation-induced chemotaxis into tumor tissue. To test this hypothesis, we modified an established permutation test (23) to assess whether receptor–ligand (RL) interactions between chemokine and receptor modules were more frequent than expected by chance. This revealed significant redundancies in possible interactions between chemokine receptor and ligand modules (Figure 1C). Chemokine module 1 interacted significantly with receptor module 4 (22 RL interactions, *P* = 0.026), which was expressed on multiple cell types (myeloid, T, NK, and B cells). Chemokine module 2 interacted mainly with receptor module 2 on granulocytes (6 RL interactions *P* = 0.0015) while chemokine modules 3 and 4 showed broad interactions with multiple receptor modules. Supporting that these candidate RL interactions were relevant for leukocyte infiltration, we found a positive correlation of chemokine module 1, 2, and 4 expression in cancer cells with the infiltration of multiple cell types of the myeloid, T, B, and NK lineages (Figure 1F). Hence, cancer cells express chemokine modules after chemoradiotherapy, which can establish redundant interactions with cognate receptor modules on immune cells and correlate with leukocyte infiltration.

### Radiotherapy induces modular SASP in a patient-derived pancreatic tumor explant model.

**Figure 2:**
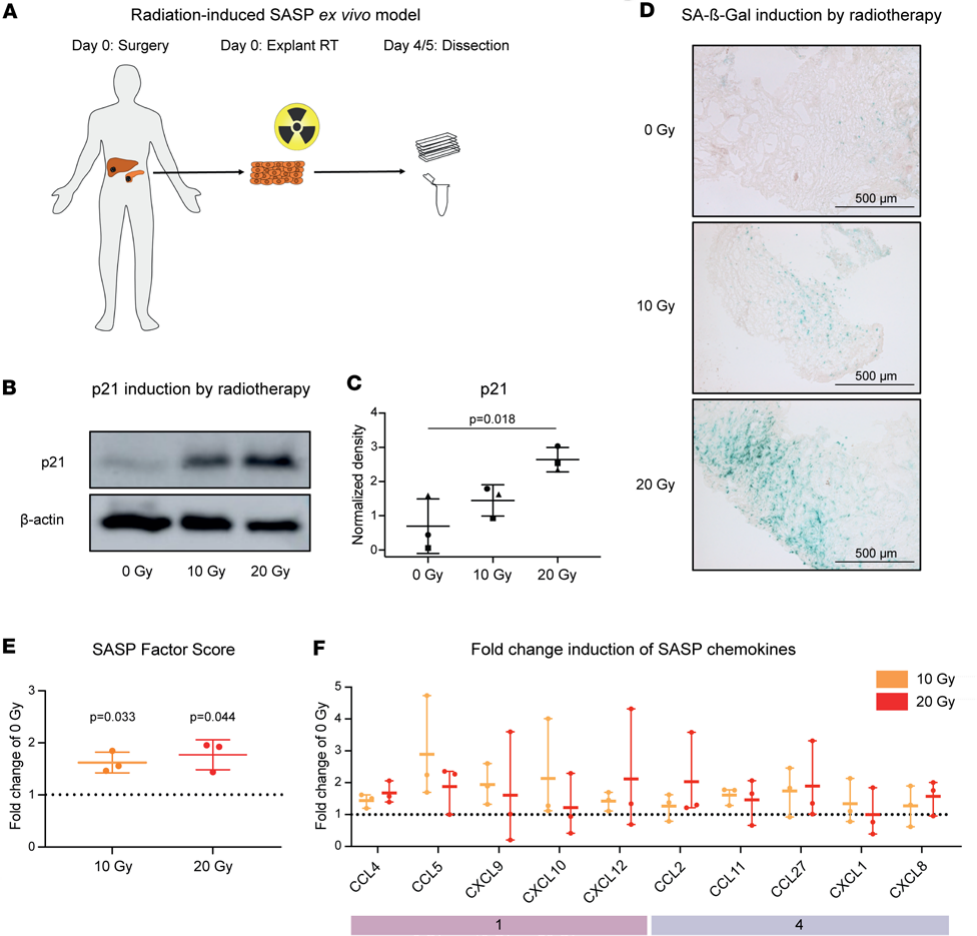
Photon irradiation induces modular SASP chemokine responses in an ex vivo pancreatic tumor model. (**A**) Schema of experimental design. (**B** and **C**) p21 is expressed in cell lysates of irradiated pancreatic cancer explants. Shown are (**B**) a representative immunoblot image and (**C**) dot plot (mean ± SD) indicating p21 density normalized to β-actin as determined in 3 independent Western blot experiments with explants from 3 patients. *P* value was calculated using unpaired *t* test. (**D**) SA-β-Gal is expressed in explants treated with 10 or 20 Gy photon radiation. Shown are representative microscopy images of SA-β-Gal and eosin staining of pancreatic cancer explant sections. (**E**) Radiotherapy induces SASP factors in explants. Dot plots showing SASP factor scores (mean fold change of 10 established SASP factors, CCL2, CCL4, CCL5, CXCL1, CXCL8, CXCL10, ICAM1, IL1a, IL6, TNFa, ± SD relative to 0 Gy) in *n* = 3 patients in 3 independent experiments. *P* values were calculated using 2-tailed 1-sample *t* tests. (**F**) Chemokines are secreted by pancreatic tumor explants after irradiation. Dot plots showing mean relative concentrations (fold change of unirradiated) ± SD of indicated chemokines (*n* = 3 patients).

Interindividual heterogeneity and the difficulty of obtaining repeated longitudinal tissue biopsies complicate studying radiotherapy-induced SASP in patients with cancer. Cell lines and organoid systems can only partially account for the complex cell type composition, which determines chemokine concentrations in the irradiated TME because of cell type–specific expression patterns (Figure 1). We therefore established a patient-derived pancreatic tumor explant model to study SASP responses to radiotherapy in tissue ex vivo. After surgical resection, pancreatic primary tumors or hepatic metastases were sectioned into thin slices and immediately irradiated with 10 or 20 Gy or left untreated (Figure 2A and Supplemental Table 2). Cellularity and tissue integrity were comparable between treated and untreated samples (Supplemental Figure 3A). Radiotherapy induced p21 expression (Figure 2, B and C, and Supplemental Figure 3B) and senescence-associated β-galactosidase (SA-β-Gal) activity (Figure 2D) as well as established SASP factors (Figure 2E, Supplemental Figure 3C, and Supplemental Table 3), suggesting potent ex vivo induction of senescence in pancreatic tumor tissue.

We next asked whether photon irradiation of our explant model induced the chemokine modules we identified above in PDAC tissue of patients who received neoadjuvant chemoradiotherapy. Irradiation with a single dose of 10 or 20 Gy homogenously induced module 1 chemokines (CCL4, CCL5, CXCL9, CXCL10, CXCL12) and module 4 chemokines (CCL2, CCL11, CCL27, CXCL1, CXCL8) (Figure 2F), while module 3 chemokines (CCL3, CCL7) remained largely unaffected (Supplemental Figure 3C).

Hence, radiotherapy simultaneously induces senescence features and modular chemokine release in primary human tumor tissue.

### Radiation induces modular chemotaxis by cancer cell–intrinsic effects.

Radiation can induce chemokines by cancer cell–intrinsic effects, such as cGAS/stimulator of interferon genes (STING) and NF-κB signaling, or through activation of tumor-infiltrating immune cells or fibroblasts sensing tumor cell death (2, 13).

To test whether cell-intrinsic effects induced modular chemokine expression after radiotherapy, we also studied radiation effects on human cancer cell lines in vitro.

**Figure 3:**
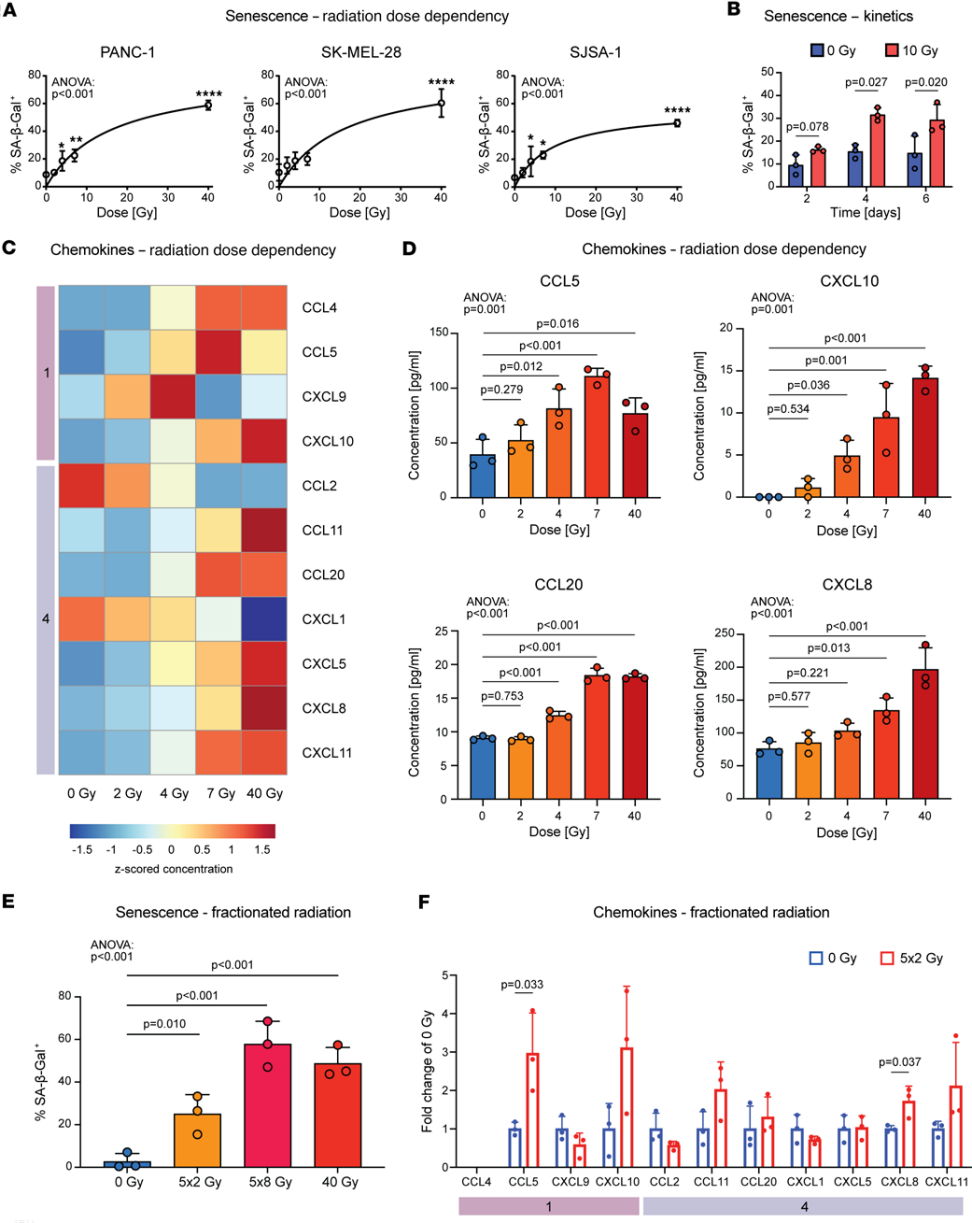
Photon irradiation induces dose-dependent senescence and chemokine release in vitro. (**A**) Photon irradiation induces the senescence marker β-galactosidase in human pancreatic cancer (PANC-1), melanoma (SK-MEL-28), and osteosarcoma (SJSA-1) cells as quantified by semiautomated microscopy (Methods). Shown is the percentage of SA-β-Gal–positive cells in 3 independent experiments (mean ± SD) 4 days after irradiation. *P* values were calculated using 1-way ANOVA, and significance was determined using the Holm-Šídák method. *P < 0.05, **P < 0.01, ****P < 0.0001. (**B**) Cellular senescence in PANC-1 cells is time dependent. Shown is the percentage of SA-β-Gal–positive cells in 3 independent experiments (mean ± SD). *P* values were calculated using paired *t* tests. (**C** and **D**) Induction of module 1 and 4 chemokines in PANC-1 cancer cells by photon irradiation is dose dependent. Chemokine concentrations in conditioned media after application of indicated radiation doses were determined in *n* = 3 independent experiments by flow cytometric bead-based immunoassays. (**C**) Shown are mean *z*-scored concentrations of indicated chemokines. (**D**) Shown are concentrations (mean ± SD) of indicated chemokines. (**E**) Fractioned radiotherapy induces cellular senescence. PANC-1 cancer cells were irradiated with indicated doses. For fractionation, cells were irradiated for 5 consecutive days. Shown is the percentage of SA-β-Gal–positive cells in 3 independent experiments (mean ± SD) on day 7 after start of radiotherapy. (**D** and **E**) *P* values were calculated with 1-way ANOVA and significance testing using the Holm-Šídák method for multiple comparisons. (**F**) Fractionated radiotherapy induces chemokine secretion. Indicated are relative chemokine concentrations in conditioned media of PANC-1 cancer cells irradiated with 0 Gy or 5 × 2 Gy normalized to control (mean ± SD). *P* values were calculated using unpaired *t* tests.

Using semiautomated microscopic quantification, we determined the dose dependencies and kinetics of cellular senescence based on SA-β-Gal staining (Methods and Supplemental Figure 4). Photon irradiation consistently induced senescence in different cancer cell types, including pancreatic adenocarcinoma (PANC-1), melanoma (SK-MEL-28), and osteosarcoma (SJSA-1) (Figure 3A and Supplemental Figure 5A). This effect was time and dose dependent, with SA-β-Gal induction starting after 4 days at doses of at least 4 Gy, with the largest effects occurring at 40 Gy (PANC-1: 6.88-fold, *P* < 0.001; SK-MEL-28: 9.17-fold, *P* < 0.001; SJSA-1: 6.85-fold, *P* < 0.001) (Figure 3, A and B, and Supplemental Figure 5). This suggests that photon radiation robustly elicits dose-dependent senescence features in cancer cell lines.

We next analyzed chemokine release from irradiated senescent cancer cells using multiplex protein assays. We found that radiation dose-dependently induced several chemokines in cancer cells, as observed in our tissue chemokine modules. Doses of at least 4 Gy induced module 1 (e.g., CCL5, CXCL10) and module 4 (e.g., CCL20, CXCL8) chemokines in pancreatic cancer cells, with larger effects using higher radiation doses (Figure 3, C and D). Senescence induction was conserved using clinically relevant fractionation protocols with 2 or 8 Gy single fractions administered for 5 consecutive days (5 × 2 Gy, 5 × 8 Gy) (Figure 3E). Correspondingly, 5 × 2 Gy increased secretion of module 1 (CCL5) and module 4 (CXCL8) chemokines by cancer cells (Figure 3F). Given the observed radiation dose dependency of senescence induction and chemokine release, we subsequently focused on high-dose radiotherapy to study leukocyte chemotaxis.

**Figure 4:**
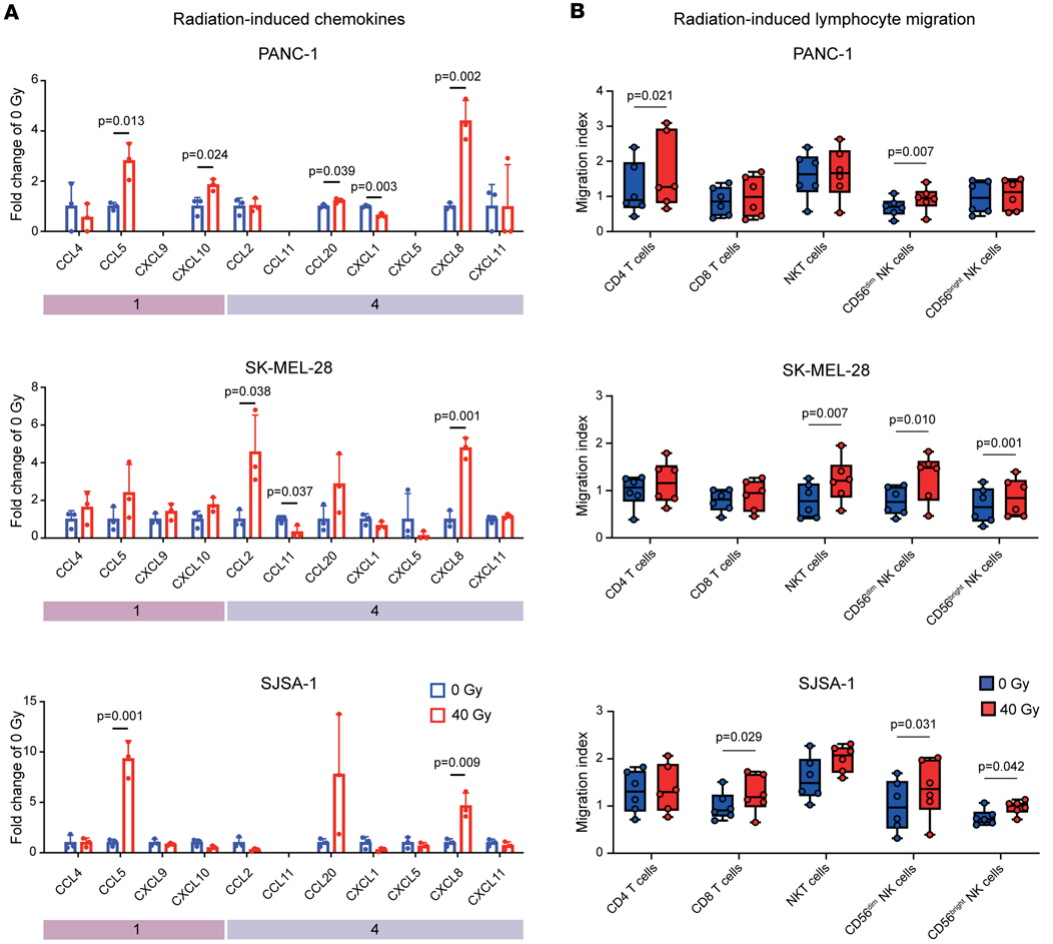
Photon irradiation–induced SASP triggers directional lymphocyte chemotaxis in vitro. (**A**) Photon irradiation (40 Gy) induces chemokine release by cancer cells as determined using a flow cytometric bead-based immunoassay. Shown are relative chemokine concentrations normalized to 0 Gy (mean ± SD). *P* values were calculated using unpaired *t* tests. (**B**) Photon irradiation of cancer cells causes human lymphocyte chemotaxis as determined by a flow cytometric modified Boyden chamber assay. Shown are migration indices (boxes indicating interquartile range, bar median, and whiskers range) of indicated immune cell types from 3 independent experiments and *n* = 6 healthy donors per cell line. *P* values were calculated using paired *t* tests.

Similar to their expression across different patients with cancer, radiotherapy with 40 Gy induced module 1 and 4 chemokines across various investigated cell lines (Figure 4A). This was most evident for CCL5 (PANC-1: 2.89-fold, *P* = 0.013; SK-MEL-28: 2.76-fold, *P* = 0.212; SJSA-1: 9.80-fold, *P* = 0.001) and CXCL8 (PANC-1: 4.43-fold, *P* = 0.002; SK-MEL-28: 5.51-fold, *P* = 0.001; SJSA-1: 4.83-fold, *P* = 0.009). Our data suggest that cell-intrinsic effects are sufficient for the induction of multiple chemokines by photon irradiation.

In patients with PDAC we found chemokine modules correlating with multiple immune cell populations, suggesting redundant chemokine receptor interactions with their cognate receptors (Figure 1). To experimentally test whether radiotherapy resulted in the simultaneous recruitment of different immune cell subsets, we established a modified Boyden chamber migration assay with primary human peripheral blood mononuclear cells (Methods and Supplemental Figure 6A). The flow cytometric readout allowed us to measure chemotaxis of 5 distinct immune cell populations in parallel. Consistent with the redundant migration patterns predicted in PDAC tissue, we observed radiation-induced migration of different T and NK cell subsets (Figure 4B and Supplemental Figure 6B). In line with the induction of CXCL8 across cancer cell lines, radiation consistently induced directional migration of CD56^dim^ NK cells (PANC-1: 1.34-fold, *P* = 0.007; SK-MEL-28: 1.66-fold, *P* = 0.010; SJSA-1: 1.36-fold, *P* = 0.031) (Figure 4B) — a cell type expressing the cognate receptors CXCR1 and CXCR2 (8, 39). Moreover, we observed higher CD56^bright^ NK cell migration toward SK-MEL-28 melanoma (1.34-fold, *P* = 0.001) and SJSA-1 osteosarcoma cells (1.27-fold, *P* = 0.042) but not toward PANC-1 pancreatic cancer cells (Figure 4B). We also detected cell line–specific chemotactic patterns: CD4^+^ T cell migration increased toward irradiated PANC-1 pancreatic cancer cells (1.39-fold, *P* = 0.021), whereas CD8^+^ T cell chemotaxis increased toward irradiated SJSA-1 osteosarcoma cells (1.26-fold, *P* = 0.029). Furthermore, NKT cells significantly migrated toward SK-MEL-28 melanoma cells (1.63-fold, *P* = 0.007) (Figure 4B).

Hence, radiation induced the simultaneous expression of several SASP chemokines by cancer cell–intrinsic effects, resulting in the chemotaxis of multiple lymphocyte subsets bearing cognate receptors.

### Radiation-induced modular chemotaxis is conserved across photon and particle radiotherapy.

Radiotherapy using charged particles, such as protons or carbon ions, is an advanced radiation treatment strategy enabling more precise physical dose distribution with higher RBE. While many effects of particle radiation have been well characterized, e.g., on clonogenic survival and proliferation (31, 40, 41), its effects on SASP and chemokine release are less well studied. Therefore, we asked whether particle and photon radiotherapy differed in SASP induction and compared both physical equivalent radiation doses as well as doses leading to similar clonogenic survival (biological equivalent radiation doses) of photons, protons, and carbon ions.

**Figure 5:**
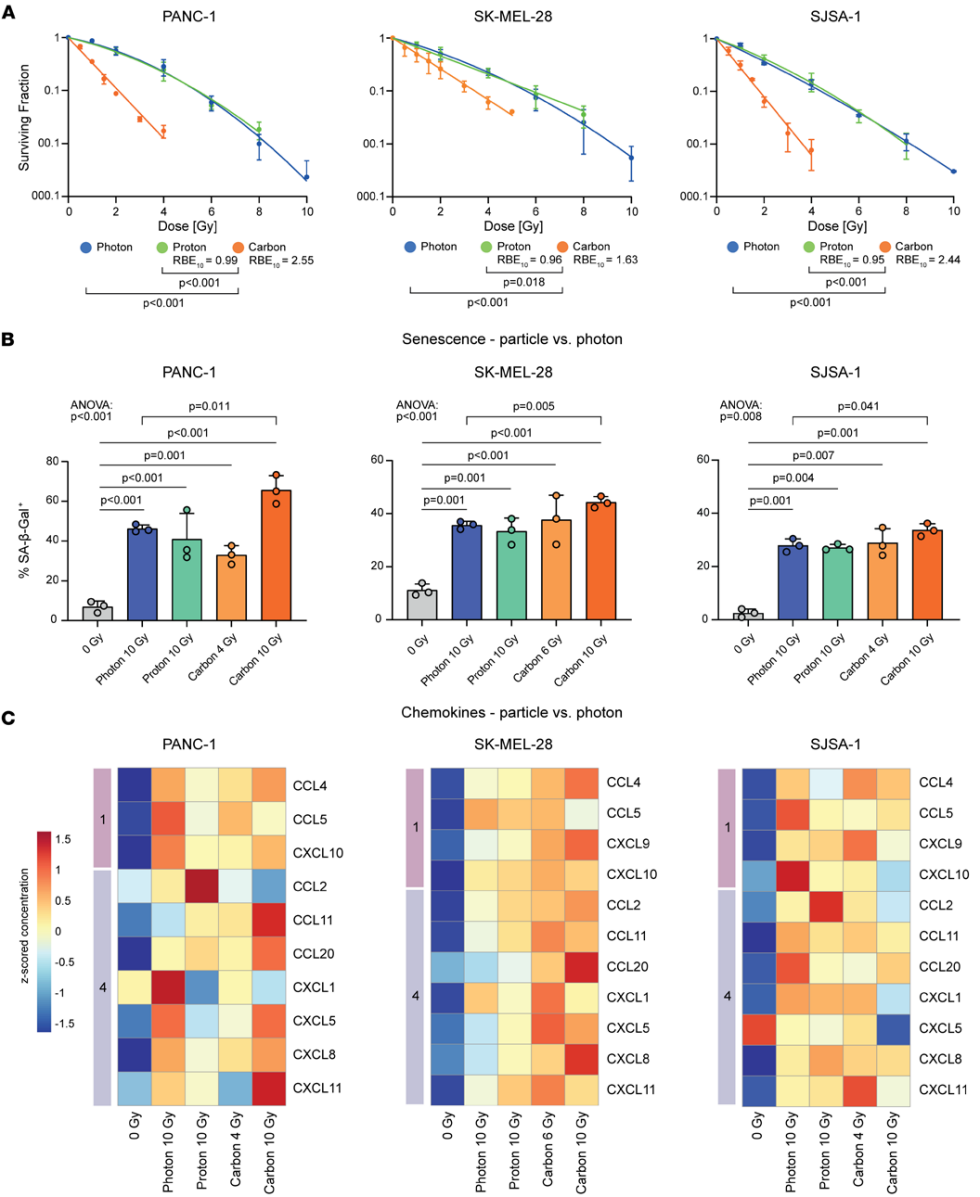
Particle irradiation induces cellular senescence and SASP. (**A**) Carbon ion irradiation inhibits clonogenic survival more strongly as compared with protons or photons. Dots indicate the surviving fraction of cells (mean ± SD) from *n* = 3 independent experiments. The connecting regression line was fitted using a linear quadratic model. *P* values were calculated using a mixed effects model. (**B**) Photon and particle irradiation induce SA-β-Gal. Shown is the percentage (mean ± SD) of SA-β-Gal–positive cells treated as indicated from *n* = 3 independent experiments. *P* values were calculated using 1-way ANOVA with determination of significance using the Holm-Šídák method or unpaired *t* tests (zig-zag line). (**C**) Photon and particle irradiation induce chemokine modules 1 and 4. Indicated are mean *z*-scored concentrations from *n* = 5 (PANC-1), *n* = 6 (SK-MEL-28), or *n* = 3 (SJSA-1) independent experiments as determined by flow cytometric bead-based immunoassays.

In line with previous studies (42, 43), physical equivalent doses of carbon ions had a higher biological effectiveness regarding clonogenic survival as compared with photons or protons (PANC-1: RBE_10_ = 2.55, SK-MEL-28: RBE_10_ = 1.63, SJSA-1: RBE_10_ = 2.44) (Figure 5A). As expected, protons did not show a significant difference in RBE when compared with photons (PANC-1: RBE_10_ = 0.99, SK-MEL-28: RBE_10_ = 0.96, SJSA1: RBE_10_ = 0.95) (Figure 5A). At the same physical dose, carbon ion irradiation induced higher rates of senescence as compared with photon irradiation (Figure 5B, Supplemental Figure 7A, and Supplemental Figure 8A), which strongly correlated with its effects on clonogenic survival (Supplemental Figure 7B). Biological equivalent doses (based on clonogenic survival RBE_10_) of carbon ions, protons, and photons induced similar rates of senescence in the investigated cancer cell lines, indicating that lower physical doses of carbon ions are sufficient to achieve the same level of senescence (PANC-1: photon 10 Gy: 7.60-fold, *P* < 0.001; proton 10 Gy: 6.40-fold, *P* < 0.001; carbon 4 Gy: 5.21-fold, *P* = 0.001; carbon 10 Gy: 11.14-fold, *P* < 0.001; SK-MEL-28: photon 10 Gy: 3.28-fold, *P* = 0.001; proton 10 Gy: 3.08-fold, *P* = 0.001; carbon 6 Gy: 3.55-fold, *P* < 0.001; carbon 10 Gy: 4.07-fold, *P* < 0.001; SJSA-1: photon 10 Gy: 15.61-fold, *P* = 0.001; proton 10 Gy: 15.70-fold, *P* = 0.004; carbon 4 Gy: 15.49-fold, *P* = 0.007; carbon 10 Gy: 19.02-fold, *P* = 0.001) (Figure 5B). Hence, carbon ions show higher biological effectiveness in inducing senescence compared with photons.

Having established the robust induction of senescence by particle irradiation, we next compared the induction of senescence-associated chemokines. At biological equivalent doses, particle and photon irradiation induced comparable release of module 1 and 4 chemokines (Figure 5C). However, physical equivalent doses of carbon ion irradiation did not substantially increase the release of most chemokines despite its much higher biological effectiveness. This suggests a plateau effect, after which dose escalation does not result in stronger chemokine release (Figure 5C). Notably, in SK-MEL-28 cells, physical equivalent doses of carbon ions resulted in a moderately higher expression of CCL20 and CXCL8 (Figure 5C and Supplemental Figure 8, B and C). This may be attributed to the greater resistance of these cells to high-dose radiation, which may have prevented them from reaching the effect plateau with 10 Gy photon radiation or biological equivalent doses of particles.

**Figure 6:**
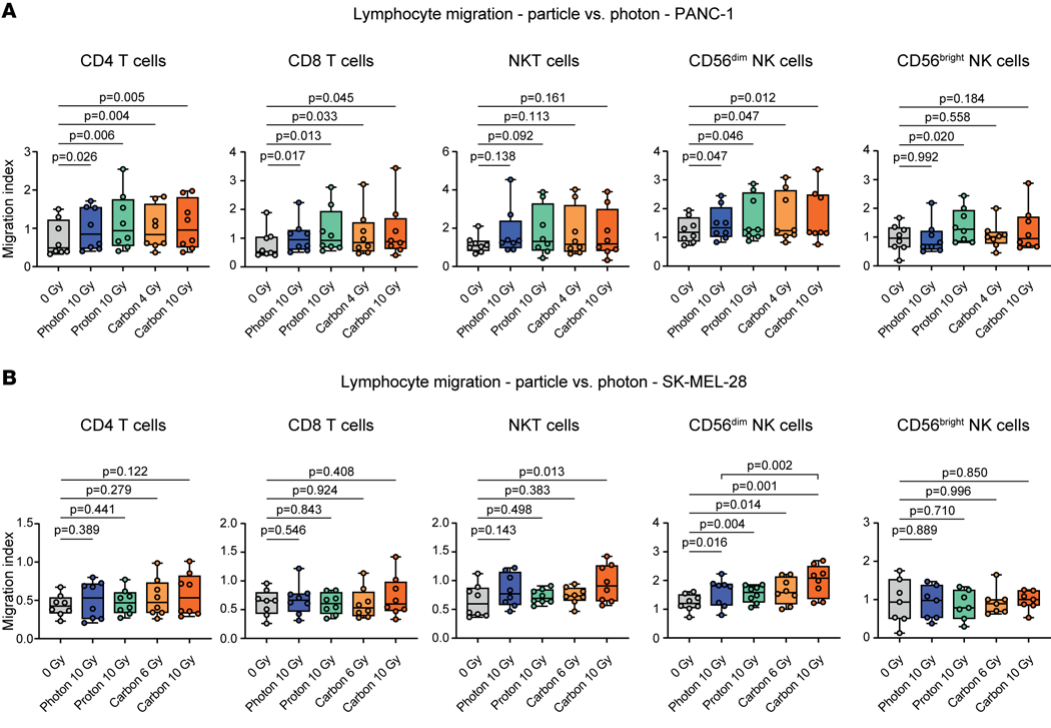
Particle irradiation induces directional lymphocyte migration. Photon, proton, and carbon ion irradiation of (**A**) PANC-1 or (**B**) SK-MEL-28 cancer cells induces lymphocyte chemotaxis in a flow cytometric modified Boyden chamber assay. Indicated are migration indices (boxes indicating interquartile range, bar median, and whiskers range) of immune cell subsets from *n* = 8 donors for each cell line from 4 independent experiments. *P* values were calculated using paired *t* tests.

Moreover, we found similar chemotactic patterns comparing photon and particle radiation–induced immune cell chemotaxis with PANC-1 and SK-MEL 28 cells using modified Boyden chamber assays (Supplemental Figure 6A). CD56^dim^ NK cells migrated toward irradiated cancer cell lines regardless of radiation type (Figure 6, A and B). Additionally, we observed comparable chemotaxis of CD4^+^ T cells and CD8^+^ T cells by PANC-1 pancreatic cancer cells after photon, proton, or carbon ion irradiation with biological and physical equivalent doses (Figure 6A). In line with its effect on CCL20 and CXCL8 chemokine release, physical equivalent carbon ion irradiation of SK-MEL-28 cells induced a moderately higher rate of CD56^dim^ NK cell migration and a significant chemotaxis of NKT cells, which we did not see after photon irradiation (Figure 6B). This is likely due to the discussed radioresistance of this cell line and suggests that the effect plateau was not yet reached with 10 Gy photon radiation or biological equivalent particle doses.

Collectively, our results suggest that modular induction of multiple chemokines is a conserved feature of radiotherapy-induced SASP across different radiation types, which is associated with chemotaxis of several lymphocyte subsets. However, radiation-induced chemokine secretion saturates at higher dose ranges, resulting in similar chemokine release and chemotactic patterns after high-dose particle or photon radiotherapy.

## Discussion

Radiotherapy effects on human tissues have not been studied systematically regarding chemokine release and recruitment of leukocyte subsets. Our analysis of single-nucleus sequencing data from irradiated pancreatic cancers revealed that radiotherapy-induced SASP chemokines are expressed in defined modules, correlating with immune cell infiltration. By introducing a potentially novel patient-derived pancreatic tumor explant model to study radiation-induced effects ex vivo, we provide experimental evidence that radiotherapy induces these SASP chemokine modules in human tissue. We show that specific chemokines from our identified modules were also induced in cancer cell lines in vitro, suggesting cancer cell–intrinsic mechanisms of radiation-induced SASP. Cognate receptors for these SASP chemokines were broadly expressed across different immune cells, and consequently, we observed chemotaxis of different lymphocyte subsets after radiotherapy. These effects were highly conserved across different types of radiation, suggesting that similar chemotactic patterns may be triggered by charged particle therapy compared with conventional photon therapy, though with a different dose-response relationship.

The modularity we observed for both SASP chemokines and their cognate receptors, paired with chemokine pleiotropy and redundancy (12, 44), results in a high number of potential interactions between cancer cells and immune cells. In line with that, SASP chemokine modules correlated with the abundance of multiple immune cell subtypes. This has important implications for strategies trying to modulate radiation-induced chemotaxis, as targeting individual chemokines may only moderately affect immune cell infiltration. Modularity also suggests that different mechanisms may regulate different SASP chemokines. Finding these mechanisms will require systematic screening efforts.

Dose and fractionation are key parameters of radiation effects, with high impact on preclinical and clinical radiotherapy studies. Here, we found that chemokine induction was generally strongest at high single doses of at least 10 Gy. However, we also observed robust and conserved SASP features across a wide range of radiotherapy protocols. Senescence and chemokine release occurred at doses as low as 4 Gy single dose and with 5 × 2 Gy conventionally fractionated protocols. Our findings are therefore relevant to both standard fractionation regimens and the higher single doses applied in SBRT. SBRT is increasingly utilized in the treatment of radioresistant tumors, such as pancreatic cancer, and has shown promising clinical outcomes (24–26, 45). Our results support previous reports that SBRT may be more effective in activating the immune system than conventional radiotherapy, partly due to reduced radiation exposure of circulating and tumor-infiltrating immune cells, while providing strong chemotactic cues (25, 46, 47).

Studying senescence and SASP in tissue is challenging, as many methods to detect senescence and SASP are primarily validated for in vitro use, and corresponding methods for the assessment of treatment effects in tissues are lacking (48). We here introduce a patient-derived pancreatic tumor explant model to allow for delivering defined radiotherapy protocols ex vivo and determined senescence according to the recommendations by the International Cell Senescence Association using a cytoplasmic marker, a nuclear marker, and SASP assessment (49). The convergence of our in vivo, ex vivo, and in vitro results suggests that the model reliably captured relevant radiobiological mechanisms.

We observed the induction of specific chemokines from our identified SASP chemokine modules in cancer cell lines, suggesting a cancer cell–intrinsic effect. The radiation-induced release of chemokines was also associated with chemotaxis of several lymphocyte subsets in vitro. In line with our previous findings (8), we found homogenous upregulation of CXCL8 after irradiation across the investigated cancer cell lines, which correlated with increased chemotaxis of NK cells. Moreover, corresponding to the significant interaction of chemokine module 1 with receptor module 4 (expressed on, e.g., T cells) in the analyzed PDAC patient data, we observed release of module 1 chemokines (CCL5 and CXCL10) by irradiated cancer cells with subsequent recruitment of T cell subsets.

Particle radiotherapy inflicts disparate DNA damage patterns compared with photon radiotherapy, resulting in stronger inhibition of clonogenic survival and therefore increased RBE (27, 30). Similarly, physical equivalent doses of carbon ion irradiation induced higher rates of cellular senescence compared with photon irradiation. However, chemokine release and chemotactic effects remained comparable. Notably, dose escalation with 10 Gy carbon ion irradiation did not substantially increase chemokine release or chemotaxis compared with 10 Gy photon irradiation. Similarly, when investigating different photon irradiation doses, we only found minor differences in chemokine release between 7 and 40 Gy. This saturation in response at higher doses suggests a plateau effect in radiation-induced chemokine induction. Determining whether this can be attributed to the saturation of radiation-induced signaling pathways such as NF-κB or cGAS/STING signaling (2, 50–53), biochemical limits in protein production, or excessive cell death at high doses warrants further investigation.

Limitations of our study include the modest sample size of the available patient data obtained from a single pancreatic cancer study, without serial biopsies and with heterogenous radiotherapy protocols. To validate our results, future studies should ideally include samples before and after radiotherapy. Similarly, our sample size of patient-derived tissue was limited, resulting in substantial variance in biological endpoints due to interindividual heterogeneity. An important challenge of this model is the substantial level of necrosis observed in pancreatic tissue. While complicating downstream analyses, necrosis is also a common cell fate in cancer (54, 55), forming part of its natural biology, especially in pancreatic tumors with high levels of digestive enzymes and tissue hypoxia (56–58). Furthermore, in this study, we link chemokine release to cellular senescence, which is a well-established connection in the literature and commonly described as part of the SASP (4, 5). However, the contribution of surviving, nonsenescent cells to chemokine release in our models is unclear. Thus, a definitive causal relationship between senescence and chemokine release at the single-cell level remains to be established. Addressing this will require overcoming technical challenges related to the detection, isolation, and processing of senescent cells, which are inherently fragile and difficult to handle. Another limitation of this study is the detection of only prespecified lymphocyte subsets in our newly established modified Boyden chamber migration assays. More diverse immune cell phenotyping to capture additional cell types will require further optimization of this method. Nevertheless, an important strength of our study is the cross-validation of our observations in patients using complementary independent experimental systems with human cancer and immune cells across a wide range of radiation doses and fractionation protocols.

In summary, our study shows that photon radiotherapy induces distinct chemokine programs with subsequent chemotaxis of lymphocytes in patients with PDAC and in ex vivo and in vitro tumor models. Our results highlight an unappreciated degree of modularity and redundancy in radiation-induced chemotaxis in human tissue. While some aspects of radiation-induced SASP are cancer cell intrinsic and can be modeled with cancer cell lines, our explant model will facilitate studying more complex SASP interactions in human pancreatic tissue.

Shaping the immune contexture of tumors in patients with cancer through radiotherapy-induced lymphocyte recruitment might facilitate immunotherapeutic approaches. Simultaneous targeting of multiple chemokines and accounting for plateau effects at high doses may be required to relevantly alter radiation-induced leukocyte migration. Hence, our findings have important implications for developing more effective radiotherapy-immunotherapy combinations in cancer.

## Methods

### Sex as a biological variable.

Both men and women were included in this study. However, we did not consider sex as a biological variable in this study, and sex-specific analyses were not performed because of the limited sample size. Given the consistency of the observed effects across samples, we believe the findings can be generalized irrespective of sex.

### Single-nucleus RNA-Seq data analysis.

Processed.h5ad files containing single-nucleus RNA-Seq (snRNA-Seq) data (*n* = 207,661) normalized to 10,000 transcripts and log1p-transformed from primary pancreatic tumors of 39 patients (35) were obtained from the NCBI Gene Expression Omnibus (GSE202051) and analyzed in python (3.10.12) using the scanpy (1.9.5) workflow. Immune cell populations were reannotated to obtain more granular and well-defined cell types. The subset of immune cells (*n* = 20,108) was obtained by partitioning the data based on the authors’ “Level 1” annotations. We filtered out 5,878 genes detected in fewer than 10 cells for a total of 16,286 genes. We obtained the 2,500 most highly variable genes using the scanpy implementation of the cell ranger method (59), which resulted in the best cell type separation after testing a range of highly variable gene numbers (2,500–7,500 genes). Because this procedure can sometimes lead to removal of important marker genes required for distinguishing important immune cell types, we used the union of these marker genes with a manually curated list of 379 marker genes, resulting in a total of 2,667 genes for downstream computation.

### Cell type annotations.

Principal components (PCs) were calculated as the smallest number of PCs explaining at least 25% of the total variance or the first 50 PCs, whichever number was higher, with a maximum of 100 PCs used. Based on this strategy, we calculated the first 50 PCs, which explained 30.96% of the total variance on the 2,667 selected genes (highly variable and marker genes). We calculated a neighbor graph (k = 15) and a UMAP embedding using the scanpy implementations scanpy.pp.neighbors and scanpy.tl.umap, respectively, with standard parameters. We clustered the data in the PC space using the scanpy implementation of PhenoGraph across a range of k parameters 10–100, selecting a k of 20, which showed high clustering stability with clusterings based on adjacent k parameters (pairwise rand index > 0.8 with k=30 and k=40) and accurately separated major immune lineages, which we annotated based on previously published marker gene signatures in the cytopus knowledge base (https://github.com/wallet-maker/cytopus) (60) Supplemental Table 1).

We then partitioned the data into the T/innate lymphoid cell (ILC), B/plasma cell, and myeloid lineage to obtain more granular annotations as described previously (8). We repeated the procedure above per lineage. For myeloid cells, we removed 1,121 further genes not expressed in myeloid cells (for a total of 15,165) genes, calculated the union of the top 5,000 most highly variable genes and marker genes (for a total of 5,021 genes for downstream analysis), calculated the first 100 PCs explaining 24.54% of the total variance, and clustered the data using PhenoGraph and a k parameter of 30, which yielded stable clustering and separated all relevant cell types. For the T/ILC lineage, we removed 2,961 genes not expressed, selected the union of the 2,500 most highly variable genes and relevant marker genes for downstream analysis (for a total of 2,553 genes), calculated the first 63 PCs explaining 25% of the total variance, and clustered the data using PhenoGraph with a k parameter of 20, which was the only parameter that accurately separated the DC subpopulations (conventional DC type 1 [cDC1], cDC2, cDC3/migratory DC). To accurately distinguish CD56^dim^ and CD56^bright^ NK cell populations, we further partitioned the data and selected only the ILCs that we clustered on the first 50 PCs (explaining 37.45% of the total variance) using a k parameter of 50. Finally, for the B/plasma cell lineage, we removed 5,742 genes present in fewer than 10 plasma/B cells. We selected the union of the 2,500 most highly variable genes and relevant marker genes (for a total of 2,522 genes) and clustered the data using a k of 30 on 62 PCs explaining 25.01% of the total variance.

Finally, we mapped the annotations obtained on the partitioned data back to the data containing all tumor-infiltrating leukocytes as well as the authors’ annotations for the nonimmune populations.

### Analyzing chemokine and chemokine receptor expression.

For all the following analyses we used only the 21 irradiated patients from this dataset (*n* = 97,987 cells). Chemokine and receptor modules were identified by calculating pairwise Spearman’s correlation coefficients for chemokine expression in cancer cells (*n* = 10,862) or chemokine receptor expression in leukocytes (*n* = 9,768). Distances between pairwise correlation coefficients were calculated using the scipy.spatial.distance.pdist function and hierarchically clustered using the scipy.cluster.hierarchy.linkage function with the “average” method. Flat clusters were extracted by the scipy.cluster.hierarchy.fcluster function using the “distance” criterion and a linkage distance of 1.65 (chemokines) or 1.5 (chemokine receptors). A module was defined as a flat cluster of gene–gene correlations with the mean pairwise Spearman’s *r* per module > 0.15. Clustering stability was assessed by a leave-one-out analysis (Supplemental Figure 1C). Specifically, we repeated the analysis after removing 1 patient from the data for each of the 21 patients. For each gene–gene pair we counted iterations where both genes fell into the same cluster and normalized this by the number of iterations.

### RL interaction permutation test.

To determine interactions between chemokines and their chemokine receptor modules, we modified an established permutation test (23). This test assessed whether the number of observed RL interactions between each pair of chemokine–chemokine receptor modules was more than expected by chance. For each chemokine and chemokine receptor module pair, we first counted RL interactions. We then randomly shuffled the chemokine and chemokine receptor labels across the modules and counted interactions. We repeated this process for 10,000 iterations to calculate a *P* value as the proportion of iterations where the random shuffled modules had an equal or greater number of interactions as compared with the real modules.

### Patient-derived pancreatic tumor explant models.

Fresh pancreatic tumor tissue from male and female patients was resected at the Department of Surgery at Heidelberg University Hospital and immediately transferred in 0.9% NaCl on ice to the laboratory for downstream processing. Transit time was generally less than 1 hour. Specimens were cut in approximately 5 mm × 5 mm × 0.5 mm slices and cultured in DMEM Glutamax (10566016, Thermo Fisher Scientific) supplemented with 1% Penicillin-Streptomycin (25030081, Thermo Fisher Scientific) solution and 10% Panexin CD (P04-930500, PAN-Biotech) on gas-permeable culture plates (150-000-362, Miltenyi Biotec). H&E staining was used to confirm histology. Samples were irradiated or left untreated and cultivated in a cell culture incubator for 4–5 days. Growth medium was exchanged daily. Tissue was then embedded and frozen in a cryopreservation matrix (41-3011-00, Medite) and stored at –80°C or formalin-fixed and paraffin-embedded. FFPE blocks were sectioned and stained with H&E. Cellularity was manually quantified through microscopy by a trained physician. Frozen tissue blocks were sectioned and protein lysates were generated for subsequent analysis (see below). All studies were performed in accordance to the Declaration of Helsinki. Patient characteristics are listed in Supplemental Table 2.

### Multiplex cytokine profiling of pancreatic tumor explants.

Quantification of cytokines was performed as previously described (32). In short, cryosections of pancreatic tumor explants were lysed using the Bio-Plex Cell Lysis Kit (171304011, Bio-Rad). Lysates were adjusted to an equal concentration, and 50 cytokines were quantified with Bio-Plex Pro human cytokine assays (Bio-Rad) according to the manufacturer’s instructions. SASP factor score was calculated after selecting 10 well-established SASP factors based on thorough literature review (Supplemental Table 3).

### Western blot.

Sections of pancreatic tumor explants were lysed using the Bio-Plex Cell Lysis Kit (171304011, Bio-Rad) according to manufacturer’s protocol. Lysates were then diluted to an equal concentration and 5 μg of protein was denatured in LDS sample buffer (NP0007, Thermo Fisher Scientific) and reducing agent (NP0004, Thermo Fisher Scientific). We loaded 30 μL of samples or 5 μL of PageRuler prestained protein ladder (26617, Thermo Fisher Scientific) on individual lanes of a Tris-glycine gel (consisting of a running gel and a stacking gel prepared using acrylamide, H_2_O, ammonium persulfate, TEMED, Tris-HCl, and SDS). Electrophoresis was run at 120 V for 100 minutes. After assembling of the blot sandwich, the blot was run for 75 minutes at 100 V on ice. Membranes were then washed and blocked for 1 hour with Tris-buffered saline supplemented with 5% BSA and 0.1% Tween 20. p21 primary antibody (2947, Cell Signaling Technology, RRID:AB_823586, 1:1,000) was added in appropriate concentrations and incubated overnight on a rotary shaker at 4°C. Then, membranes were washed and incubated with horseradish peroxidase–coupled goat polyclonal anti-rabbit secondary antibody (7074, Cell Signaling Technology, RRID:AB_2099233), (1:2,000) for 1 hour, then incubated in LumiGlo solution (7003S, Cell Signaling Technology) for 2 minutes, and luminescence was documented using an Amersham Imager 680 (General Electric). Membranes were stripped and the procedure was repeated using β-actin primary antibody (4967, Cell Signaling Technology, RRID:AB_330288, 1:2,000). For subsequent analysis, density of protein bands was quantified using ImageJ (v1.52d) with normalization to β-actin loading control.

### Cell culture.

Human tumor cell lines were obtained from the American Type Culture Collection and cultured in RPMI (A1049101, Thermo Fisher Scientific) supplemented with 10% fetal bovine serum (S181B-500, Biowest), 2 mM l-glutamine (25030081, Thermo Fisher Scientific), and 1% Penicillin-Streptomycin solution (P4333, Merck). Cells were cultured at 37°C with 5% CO_2_ and passaged every 2–3 days. Before any experiment, cells were detached and transferred to new cell culture flasks (690175 /658175/660175, Greiner Bio One) 2–3 days earlier and harvested in log growth phase.

### SA-β-Gal assay and semiautomated quantification of senescence.

To comprehensively assess the capacity of photon radiation to induce senescence in cancer cells, we established an unbiased, semiautomated macro-based quantification approach based on SA-β-Gal staining. For in vitro experiments cells were irradiated at indicated doses or left untreated and transferred to 24-well plates containing sterile microscopy coverslips. After 4 days, cells were stained for SA-β-Gal using a dedicated staining kit (9860S, Cell Signaling Technology) as per manufacturer instructions. In short, cells were washed with PBS and fixated for 10–15 minutes. Then, after rinsing the plates with PBS, cells were incubated in β-Galactosidase staining solution at pH 6 overnight at 37°C. pH was determined using a pH meter (pH meter 766, Knick) and adjusted with HCl or NaOH if needed. Next, after washing, nuclei were stained with DAPI (6335.2, Carl Roth) by incubation of cells in a 1 μg/mL DAPI in H_2_O solution for 45 minutes. After immersion in H_2_O, coverslips were mounted on microscopy slides using Fluoromount-G (0100-01, SouthernBiotech). Images were visualized using a motorized inverted Axio Observer Z1 microscope (Carl Zeiss) equipped with a 365 nm LED Colibri excitation source and fluorescence filter for DAPI (62HE Carl Zeiss filter set) and transmitted light for bright-field images of SA-β-Gal staining using a 10×/0.3 Plan-NEO Ph1 DICIII objective. Five corresponding random images per condition of DAPI and SA-β-Gal staining were acquired sequentially with the same 12-bit AxioCam MRc color charge-coupled device camera. The microscope was operated using ZEN 2012 (blue edition) software.

Images of SA-β-Gal and DAPI costained cells were analyzed using an ImageJ (v1.52d) (NIH) macro that counts the total cell number based on DAPI-positive nuclei and quantifies the amount of β-galactosidase–positive cells by measuring color intensity maxima in bright-field images. The macro and a detailed manual are provided elsewhere (https://github.com/WALL-E-Lab/senescence-quantifier) (doi: 10.5281/zenodo.16751452). In short, DAPI images are opened and settings (Gaussian blur radius, minimum threshold, prominence, minimum positive cell area) for segmentation are chosen. Background from DAPI images is subtracted using Rolling Ball Background Subtraction. Images are then filtered by Gaussian Blur with the selected radius. Subsequently, segmentation is conducted through the Find Maxima tool with the chosen values for prominence and minimum threshold. Then, particles are analyzed and cell count is displayed. Corresponding bright-field images of SA-β-Gal–stained cells are opened and RGB color areas (SA-β-Gal signal) in proximity of DAPI signals are identified. For this, background from SA-β-Gal images is subtracted using the Rolling Background Subtraction, and Gaussian Blur with selected radius is applied for filtering. Then, images are segmented using the Find Maxima tool with the determined prominence and minimum threshold. Through enlarging of DAPI areas and subsequent analysis being limited to resulting areas, it is assured that only SA-β-Gal signal in proximity to nuclei is detected. Last, regions of interest above the entered minimum area are quantified and results are printed. The relative number of senescent cells was calculated by dividing the number of SA-β-Gal–positive cells by the number of total cells as determined by nuclei quantification.

The macro reliably identified senescent cells and was sufficient to quantify senescence in irradiated cells and untreated controls (Supplemental Figure 4A). The results of the macro-based approach correlated well with the standard method of manual quantification (*R*^2^ = 0.9689) with the advantage of objectivity and higher throughput (Supplemental Figure 4, B and C).

For SA-β-Gal staining in pancreatic tumor explants, frozen sections of tumor tissue were thawed, and staining was performed with the SA-β-Gal staining kit (9860S, Cell Signaling Technology) as described above. Subsequently samples were counterstained with eosin before image acquisition.

### Irradiation.

For photon radiation treatment, cells were irradiated in suspension with a Cesium-137 source using a Gammacell 1000 device (Atomic Energy of Canada Ltd.) and then transferred into cell culture flasks. Human pancreatic tumor explants and cells for the experiments in Figures 5 and 6 were treated using a MultiRad 350 x-ray irradiation system (Faxitron).

For particle irradiation, protons and carbon ions were generated in the patient treatment–grade HIT cyclotron. Ion beams were delivered with raster scanning technique in a horizontal beamline with an 8 mm spread-out Bragg peak that was adjusted using a 30 mm water-equivalent range shifter. For protons, energy levels of 64–70 MeV/u with an average linear energy transfer of ~5.1 keV/μm were used. For carbon ions, energy levels of 123–137 MeV/u with an average linear energy transfer of ~100 keV/μm were used. Dosimetry was performed on a routine basis by medical physicists.

### Bead-based multiplex protein assays.

For the quantification of soluble proteins in the supernatants of cancer cells, LEGENDplex multiplex bead-based assays (740985, BioLegend) were used according to the manufacturer’s instructions. Briefly, supernatants were incubated with antibodies coupled to fluorescence-encoded beads and detection antibodies in 96-well, V-bottom, low–protein-binding polypropylene microplates (651201, Greiner Bio One). Subsequently, staining with Streptavidin-PE was performed, and beads were analyzed on a flow cytometer as described below. Concentrations were interpolated from a standard curve using 5-parameter logistic regression calculated with LEGENDplex 8.0 Software (BioLegend).

### Isolation of human PBMCs.

Fresh buffy coats from male and female healthy blood donors were acquired through Deutsches Rotes Kreuz DRK-Blutspendedienst Baden-Württemberg-Hessen gGmbH (Mannheim, Germany). All studies were performed in accordance with the Declaration of Helsinki. Human PBMCs were separated from buffy coats using density gradient centrifugation with a polysucrose separating solution (L6115, Biochrom) and immediately used for downstream experiments.

### PBMC migration assay.

Leukocyte migration toward conditioned media of irradiated or nonirradiated cancer cells was assessed using a modified Boyden chamber assay. Cell-free conditioned media of treated or untreated cancer cells were collected 5 days after irradiation. At 24 hours before collection of supernatants, growth medium was changed into RPMI containing 0.5% BSA. Cells and fragments were removed from supernatants by repeated centrifugation and filtration using a PVDF Filter (P666.1, Carl Roth). Cell-free conditioned media were then filled in the lower well of Transwell migration plates (3421, Corning) as per manufacturer’s instructions. Human PBMCs were freshly isolated as described above and placed in the upper chamber. Cells were then incubated for 4 hours at 37°C in a cell culture incubator. Migrated cells in the lower chamber were harvested and stained for surface antigens as described below. Quantification was done using flow cytometry. For cell counting equal numbers of Accudrop beads (345249, BD) were added to each sample, and identical numbers of beads were acquired per sample. All samples were gated for singlets, 7-aminoactinomycin D (7AAD) negativity, and subset-specific markers: CD4^+^ T cells (CD3^+^CD4+), CD8^+^ T cells (CD3^+^CD8^+^), NK T cells (CD3^+^CD56^+^), CD56^dim^ NK cells (CD56^dim^CD16^+^), and CD56^bright^ NK cells (CD56^bright^CD16^–^). Migration indices were calculated by dividing the number of cells that migrated to the lower chamber containing the test sample by the number of migrated cells in the negative control sample with plain growth medium in the lower chamber.

### Flow cytometry.

For flow cytometry, cells were transferred to 96-well, U-bottom plates (92697, TPP) and washed repeatedly with flow cytometry buffer (PBS + 3% FBS and 0.05% NaN_3_). Antibody staining mixes were prepared using flow cytometry buffer supplemented with 10% human blood group AB serum (H3667, Merck) and the following antibodies: CD3-PE/Cy7 (RRID:AB_2561451, OKT3, 317333, BioLegend, conc.: 0.625 μg/mL), CD4-FITC (RRID:AB_395751, RPA-T4, 555346, BD, dilution: 1:10), CD8-PacificBlue (RRID:AB_10551616, SK1, BioLegend, 344717, conc.: 1.25 μg/mL), CD14-PE (RRID:AB_395799, M5E2, 555398, dilution 1:10), CD16-APC/Cy7 (RRID:AB_2562952, B73.1, 360709, BioLegend, conc.: 5 μg/mL), and CD56-APC (RRID:AB_604106, HCD56, 318310, BioLegend, conc. 50 μg/mL). Used antibody concentrations and dilutions were determined in titration experiments with human PBMCs to discriminate positive from negative populations while determining background staining as assessed by isotype controls. Cells were incubated with the antibodies on ice for 30 minutes. After subsequent washing in flow cytometry buffer, 7AAD (420404, BioLegend) was added to each sample at 1:40 dilution and incubated on ice for 5 minutes for live/dead discrimination. Samples were then transferred to 5 mL polystyrene tubes (352008, Corning) and acquired on a BD FACSCanto II flow cytometer with BD FACS DIVA v.8.0 software. Data analysis was performed using FlowJo_v10.8.0 (FlowJo LLC).

### Clonogenic survival assay.

To determine differences in radiosensitivity of cancer cells to irradiation with photons, protons, or carbon ions, clonogenic survival assays were performed. For this, cells were irradiated with different doses of different radiation types, and colony formation was assessed after 6–8 days. Cells were then fixed with 33% acetic acid in methanol before staining with crystal violet. Colonies with more than 50 cells were counted and surviving fractions calculated. The linear quadratic model was applied to fit survival curves using SigmaPlot v13. To estimate biological equivalent doses of particles, the RBE at 10% clonogenic cell survival (RBE_10_) was used as previously described (61, 62).

### ELISA.

For the quantification of CXCL8 in conditioned media, ELISA (DY208, R&D Systems) was performed according to the manufacturers’ instructions. In brief, the capture antibody was incubated on 96-well, flat-bottom microplates (655061, Greiner Bio One) overnight. Then, samples and standards were loaded after plate blocking with a buffer containing 1% BSA. After incubation with the detection antibody, streptavidin-HRP was added. The color reaction was started with TMB substrate solution (T0440, Merck) and stopped with H_2_SO_4_. Absorbance was measured using an Infinite 200 PRO microplate reader (Tecan).

### Statistics.

snRNA-Seq data were analyzed as described above. Statistical analysis of ex vivo and in vitro studies was performed using Prism 8.4 (GraphPad Software). *P* values were calculated as indicated in the figure legends using 2-tailed *t* tests, 1-way ANOVA, or mixed effects model. In the case of ANOVA, the Holm-Šídák method for multiple comparisons was applied. *P* values less than 0.05 were generally considered statistically significant.

### Study approval.

Human pancreatic tumor tissue was obtained after written informed consent as approved by the institutional review board at Heidelberg University (Ethikkomission I, Heidelberg University, approval number: S-457/2019). Fresh buffy coats from male and female healthy blood donors were acquired through Deutsches Rotes Kreuz DRK-Blutspendedienst Baden-Württemberg-Hessen gGmbH (Mannheim, Germany), and written informed consent was obtained from all participants. Institutional review board approval was obtained from Ethikkomission I (S-308/2025), Heidelberg University, Medical Faculty Heidelberg, Heidelberg, Germany, and Ethikkomission II (87/04), Heidelberg University, Medical Faculty Mannheim, Mannheim, Germany.

### Data availability.

The analyzed snRNA-Seq data in this study were obtained from the Gene Expression Omnibus at GSE202051. The source code used in this study is publicly available on GitHub at https://github.com/wallet-maker/2023_Particle_SASP (commit ID 82836c7) (doi: 10.5281/zenodo.16751444) and https://github.com/WALL-E-Lab/senescence-quantifier (commit ID 72cd163) (doi: 10.5281/zenodo.16751452). Values for all data points in each graph are reported in the Supporting Data Values file. Any other information is available upon request from the corresponding authors in compliance with ethics approvals regarding patient information.

## Author contributions

The project was conceptualized by JAK, PEH, and TW. Investigation was conducted by JAK, MMA, AS, BL, AA, MSC, MH, DK, ATD, TB, JMS, DA, TT, ML, RLP, and TW. Methodology was developed and implemented by JAK, BL, AA, MSC, DK, SB, NAG, CG, RLP, DJ, JD, NH, PEH, and TW. Data curation, formal analysis, and visualization were done by JAK and TW. Resources were provided by NAG, DJ, JD, NH, PEH, and TW. Funding acquisition was carried out by JAK, PEH, and TW. The original draft was written by JAK, JL, PEH, and TW. All authors contributed to review and editing of the manuscript.

## Supplementary Material

Supplemental data

Unedited blot and gel images

Supplemental table 1

Supporting data values

## Version 1 08/14/2025

In-Press Preview

## Version 2 09/23/2025

Electronic publication
