# Supplementary material for: Photon and particle radiotherapy induce redundant modular chemotaxis of human lymphocytes
Source: JCI Insight. 2025 Aug 14;10(18):e190149. doi: 10.1172/jci.insight.190149 (PMC12487834; doi:10.1172/jci.insight.190149)
Supplement: Supplemental data [file jciinsight-10-190149-s244.pdf]

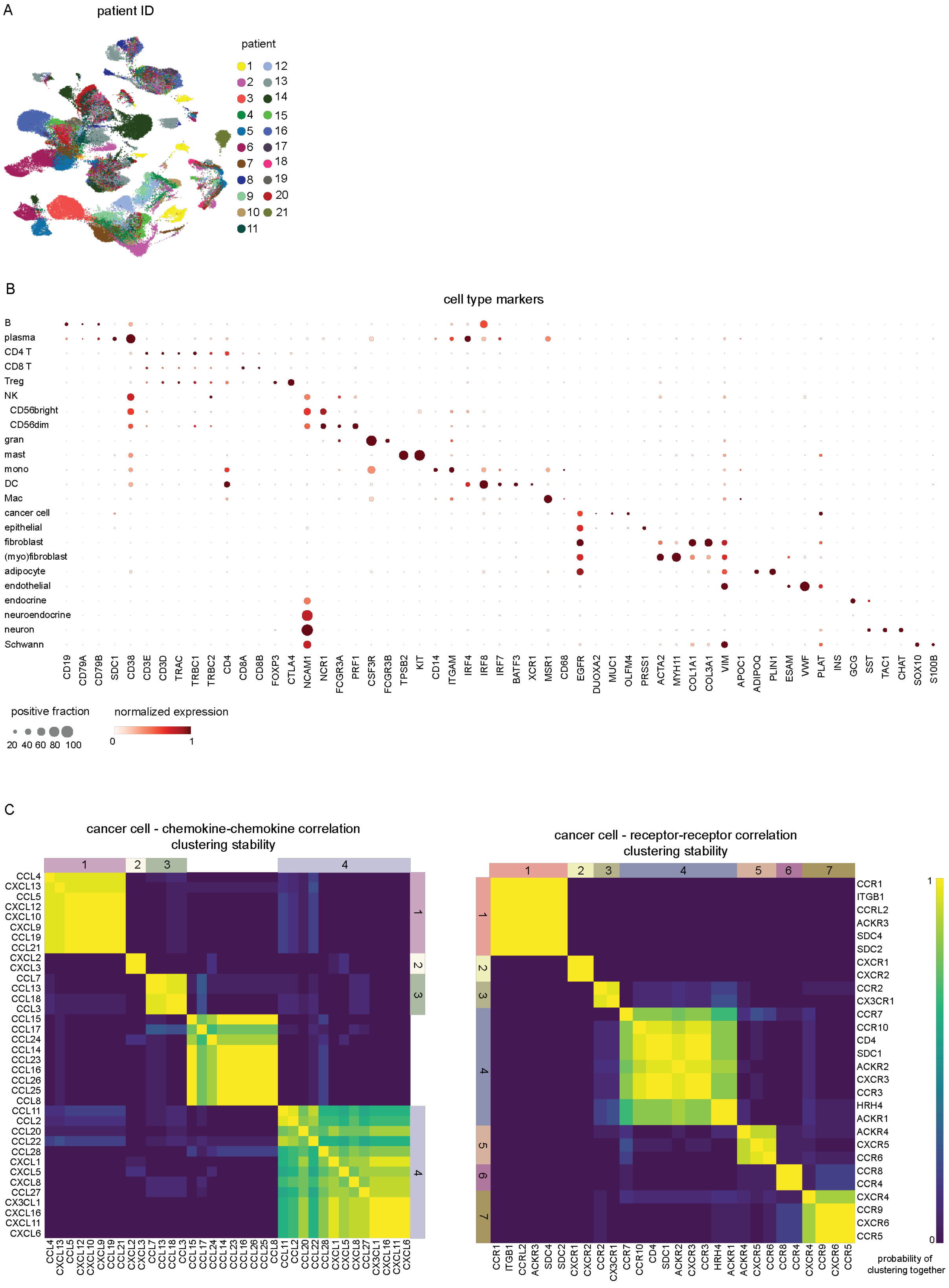

Supplemental Figure S1. Cell type annotations and clustering stability of snRNAseq data, related to Figure 1A. **A,** UMAP of snRNAseq data from pancreatic cancers after chemoradiotherapy from Hwang et al. (Nature Genetics, 2022) with color code indicating the patient (n=97,987 cells). **B,** Normalized cell type marker expression (per gene) for relevant cell types in the data. Immune cells were re-annotated as outlined in Methods. **C,** Stability of hierarchical clustering as determined by leave-one-out analysis (Methods). Indicated is the probability of two chemokines (left panel) or two chemokine receptors (right panel) falling into the same cluster.

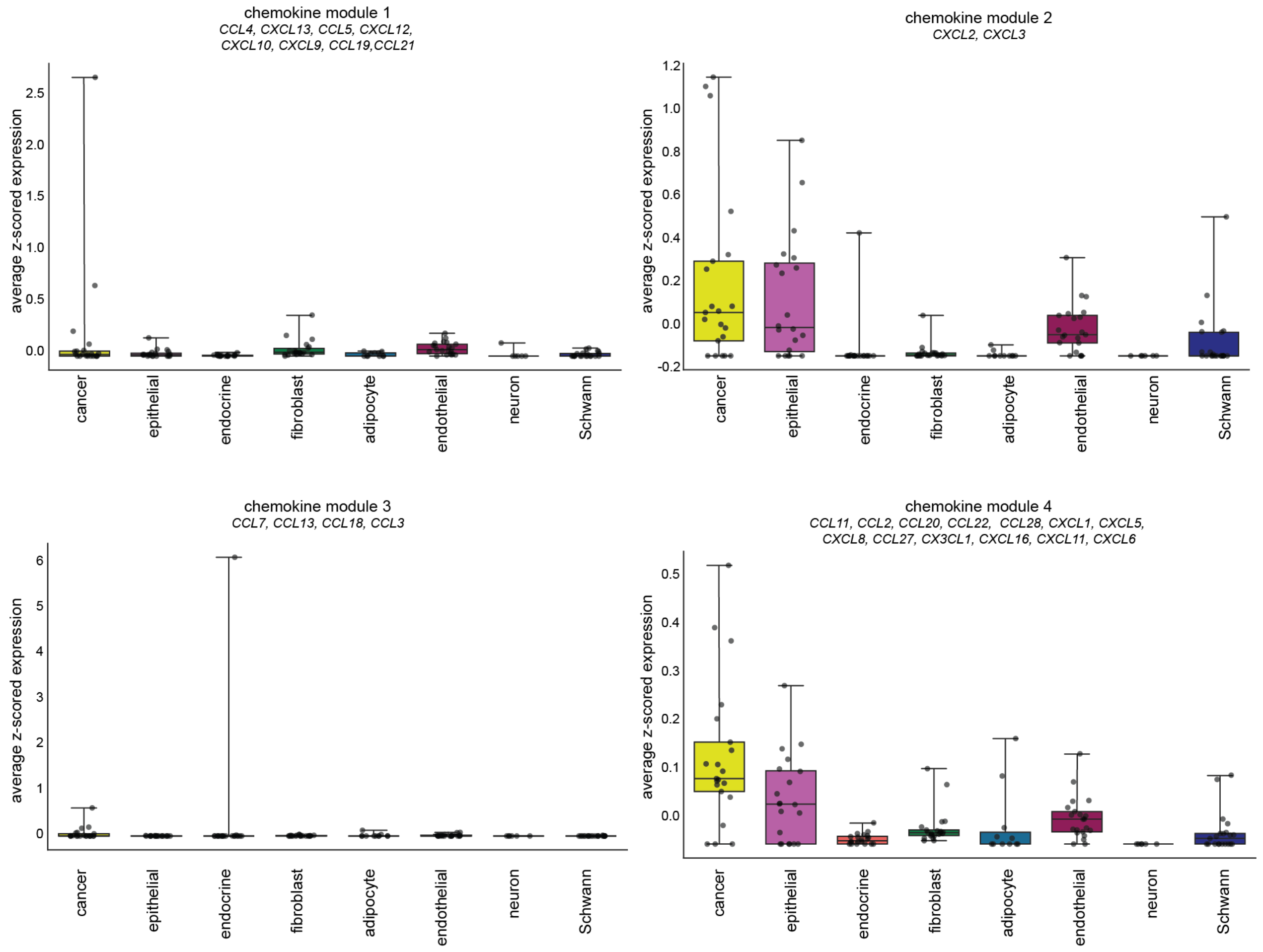

Supplemental Figure S2. Chemokine module expression across cell types. Related to Figure 1. Average z-scored (across cells) chemokine expression per patient sample (n=21) and cell type was calculated. Boxes indicate interquartile range (not considering outliers), whiskers range and bar median. Outliers are determined as datapoints above the third quartile + 1.5x interquartile range or below the first quartile - 1.5x interquartile range.

A

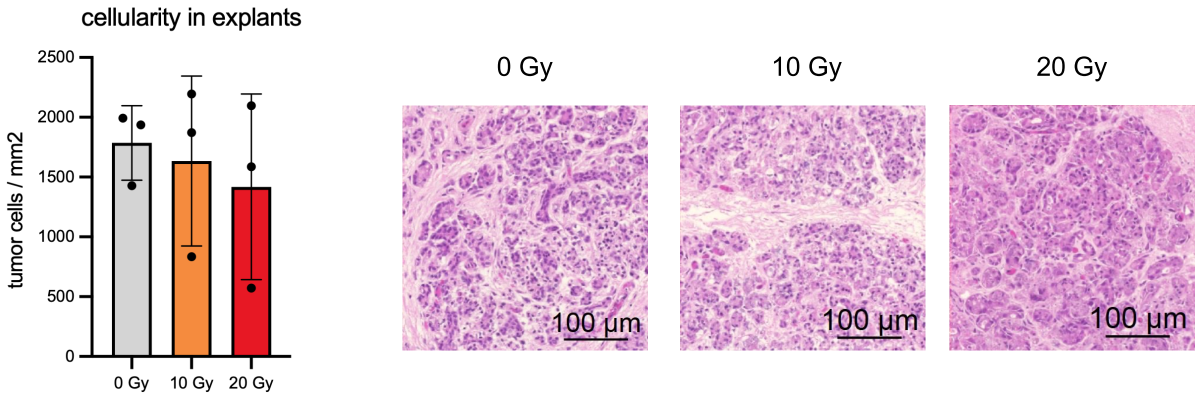

B

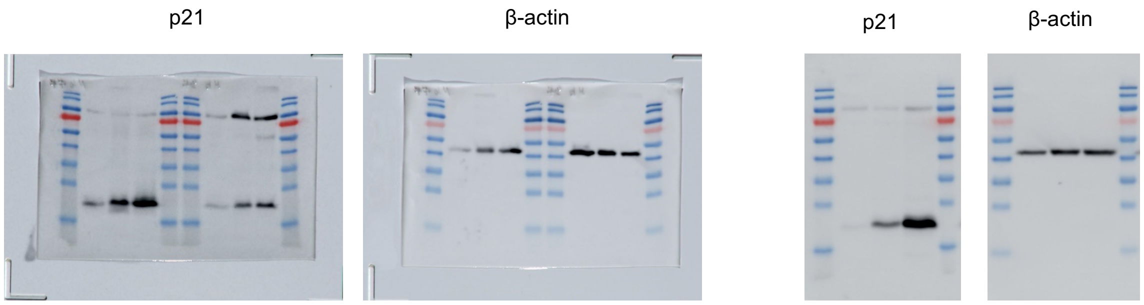

C

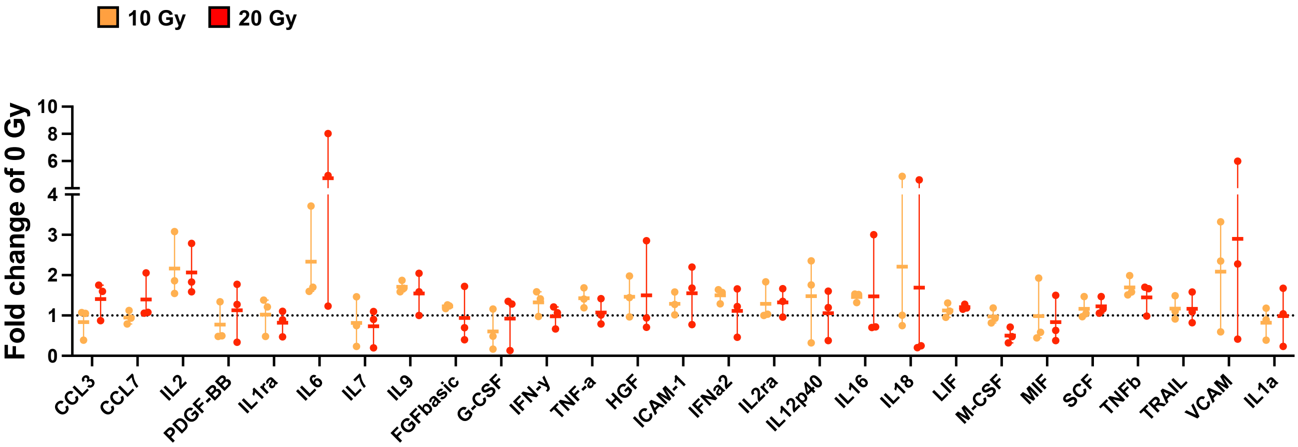

Supplemental Figure S3. p21 and cytokine expression in patient-derived pancreatic tumor explants. Related to Figure 2. **A,** Quantification of tumor cells in pancreatic tumor explants. Left Panel: bar graphs indicating tumor cells / mm^2 as quantified by H&E staining of FFPE tissue 4 days after treatment with indicated doses. Right panel: representative H&E microscopy images **B,** Shown are full length original immunoblot images for p21 and β-actin expression in pancreatic tumor explants treated with 0 Gy, 10 Gy or 20 Gy. Lanes from left to right: patient #1 0Gy, patient #1 10Gy, patient #1 20Gy, patient #2 0Gy, patient #2 10Gy, patient #2 20Gy (left panel), patient #4 0Gy, patient #4 10Gy, patient #4 20Gy (right panel). **C,** Expression of cytokines in pancreatic tumor explants after irradiation. Tumor explants of 3 pancreatic cancer patients were irradiated with indicated doses in 3 independent experiments. 5 days after treatment, cytokine secretion was analyzed using a multiplex cytokine assay. Indicated are dot plots showing relative concentrations (fold change of unirradiated) of indicated cytokines.

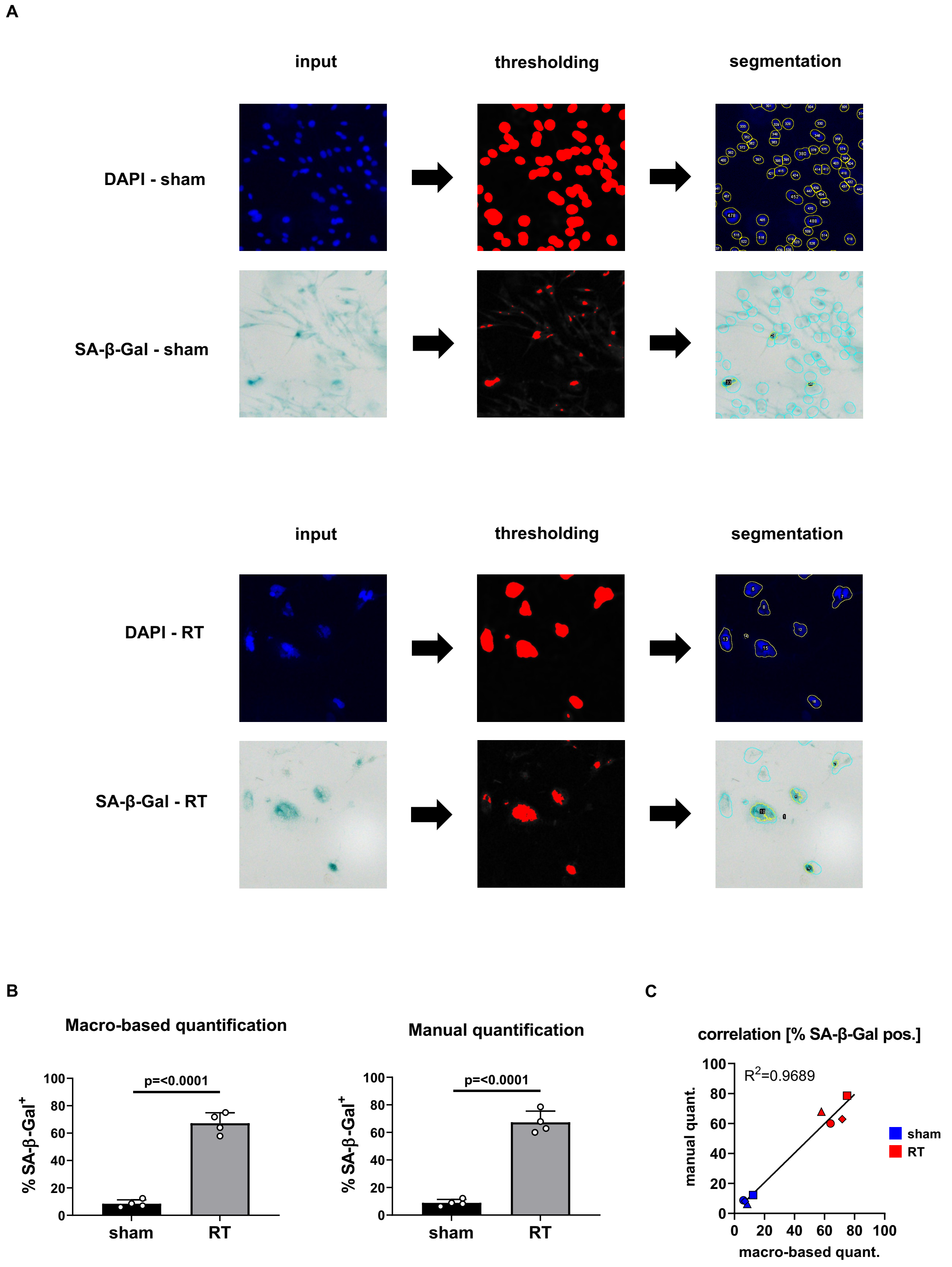

Supplemental Figure S4. Macro-based quantification of radiation-induced cellular senescence. Related to Figure 3. **A-C,** SK-MEL-28 human melanoma cells were left untreated (sham) or irradiated with 40 Gy (RT). 4 days later, cells were stained for SA-β-Gal and DAPI. Subsequently, images were acquired on a fluorescence microscope in DAPI and brightlight channel. **A,** Representative details of images. Input images were processed via thresholding and automatic segmentation as indicated during macro-based quantification. **B**, Indicated is the percentage of SA-β-Gal cells of four random high power microscopy fields for each condition. Left panel: SA-β-Gal positive cells and total cells were quantified using macro-based image analysis. Right panel: Images were quantified by manual cell counting. P-values were calculated using unpaired t-test. **C**, Scatter dot blot of percentage of SA-β-Gal positive cells as quantified by indicated method. R^2 was calculated using linear regression.

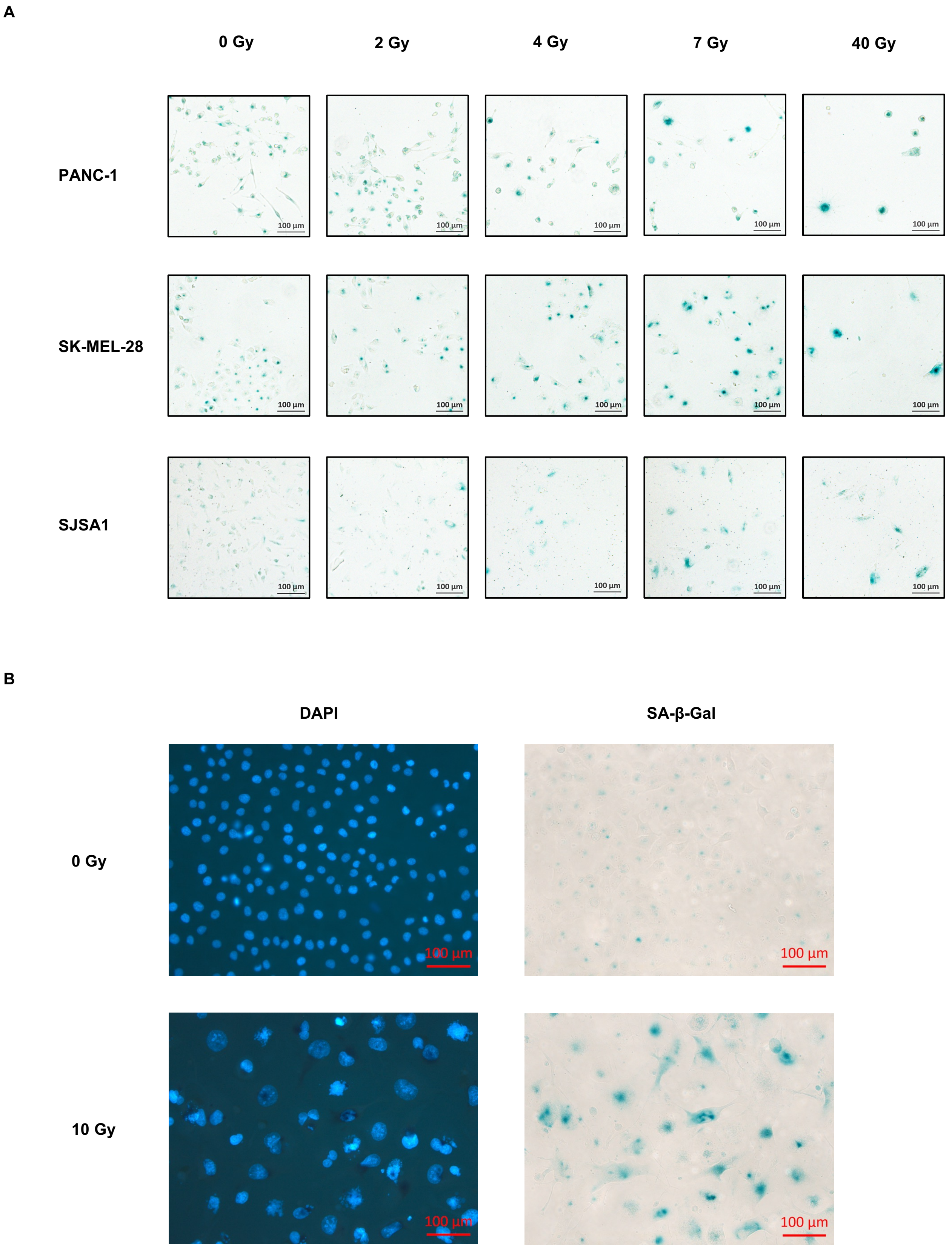

Supplemental Figure S5. A, Dose-dependent induction of senescence by photon irradiation. Related to Figure 3. Shown are representative images of SA-β-Gal stained cancer cells irradiated with indicated doses. **B,** Shown are high magnification representative images of SA-β-Gal and DAPI stained PANC-1 cancer cells irradiated with indicated doses at day 4 after treatment.

A

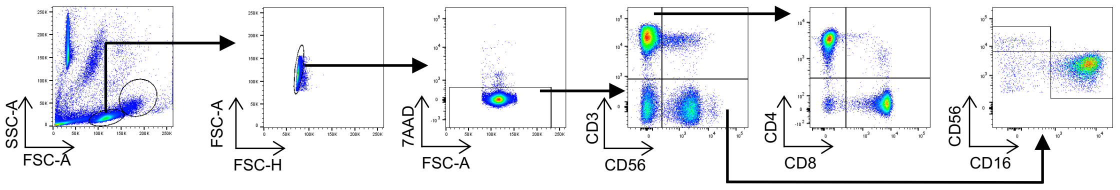

B

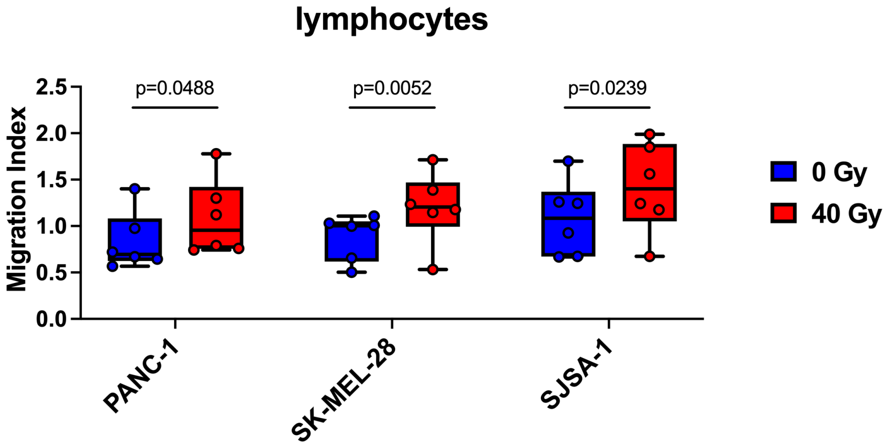

Supplemental Figure S6. Radiation-induced chemotaxis of lymphocyte subsets**.** Related to Figure 4. Freshly isolated PBMC were allowed to migrate towards conditioned media of irradiated or untreated cancer cells. Migrated cells were then analyzed by flow cytometry. All samples were gated for singlets, 7AAD- and cell specific markers: CD4-T-Cells (CD3+, CD4+), CD8-T-Cells (CD3+, CD8+), NKT-Cells (CD3+, CD56+), CD56dim-NK-Cells (CD56dim, CD16+), CD56bright-NK-Cells (CD56bright, CD16-), Monocytes (CD14+/CD16+). **A,** Representative gating strategy. **B,** Indicated are the migration indices (median +/- range and individual data points) of migrated investigated lymphocytes (defined as the sum of CD4-T-Cells (CD3+, CD4+), CD8-T-Cells (CD3+, CD8+), NKT-Cells (CD3+, CD56+), CD56dim-NK-Cells (CD56dim, CD16+) and CD56bright-NK-Cells (CD56bright, CD16-), For each cell line 3 independent experiments with PBMC from 6 healthy donors were performed with each dot representing an individual donor. P-values were calculated using paired t-tests.

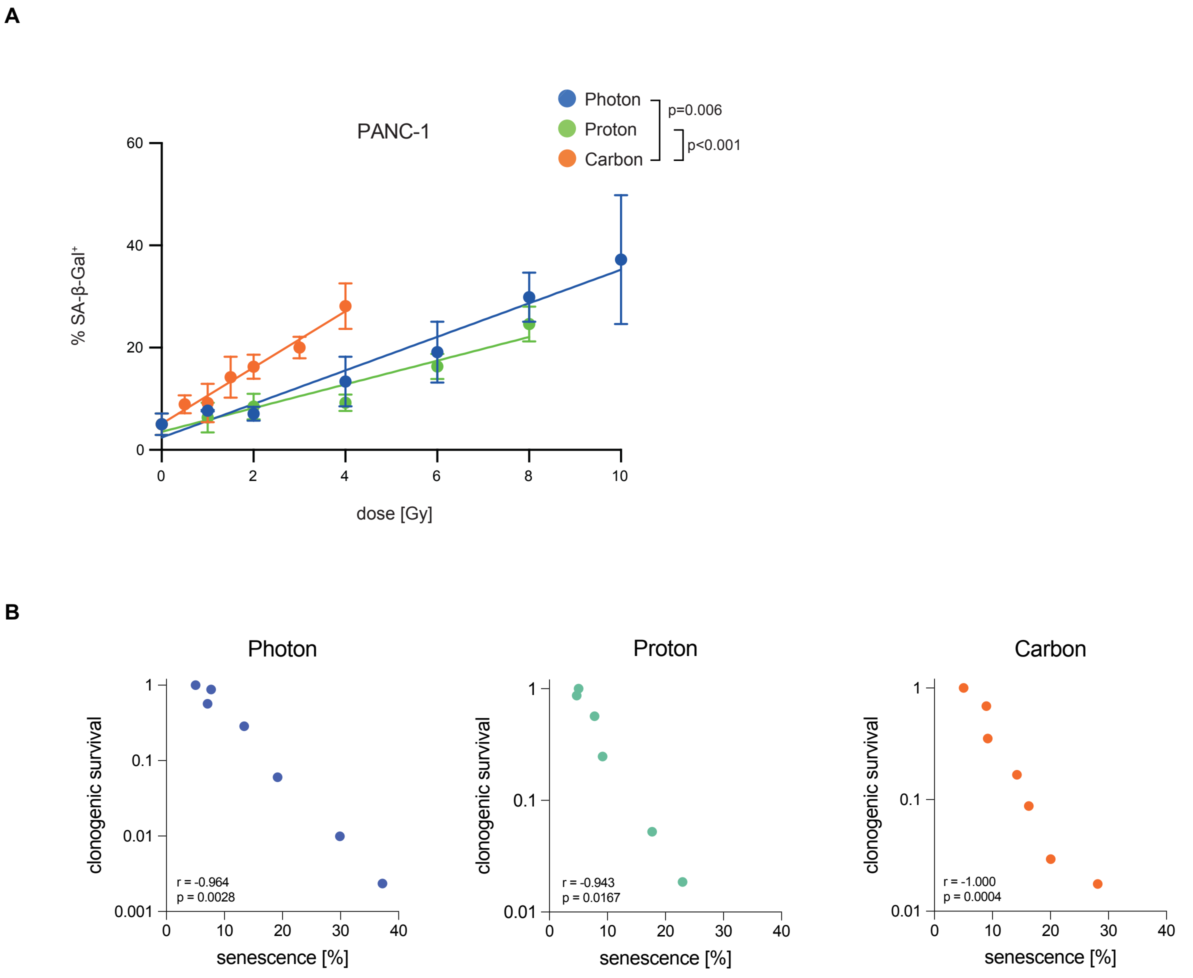

Supplemental Figure S7. Photon and particle irradiation induces dose-dependent cellular senescence which correlates strongly with clonogenic survival outcomes. Related to Figure 5. **A,** Shown is the percentage (mean ± SD) of SA-β-Gal positive PANC-1 cancer cells, which were treated with the indicated doses of photon, proton or carbon ion irradiation. The regression line was fitted with linear regression. P-values were calculated using a mixed effects model. **B,** Clonogenic survival and senescence show a strong (negative) correlation in all investigated radiation types. Clonogenic survival was measured by clonogenic survival assays as indicated in Figure 5A. Senescence was measured by SA-β-Gal assays as indicated in Figure S7A. Correlation was calculated using Spearman correlation.

A

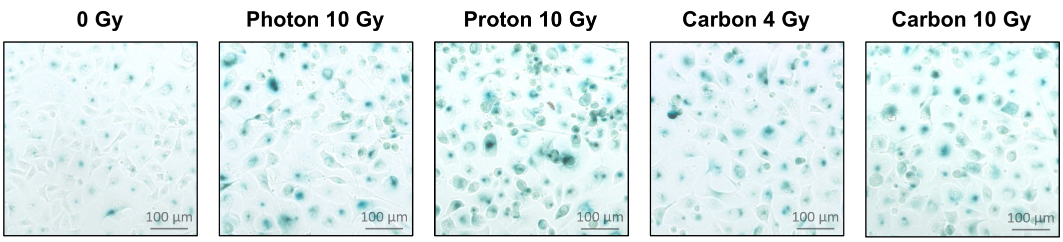

B

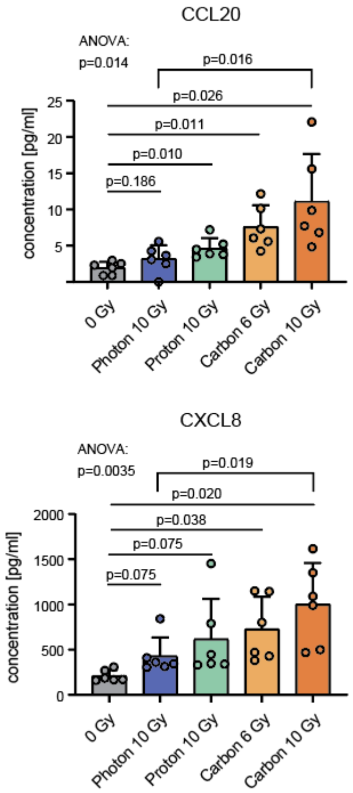

C

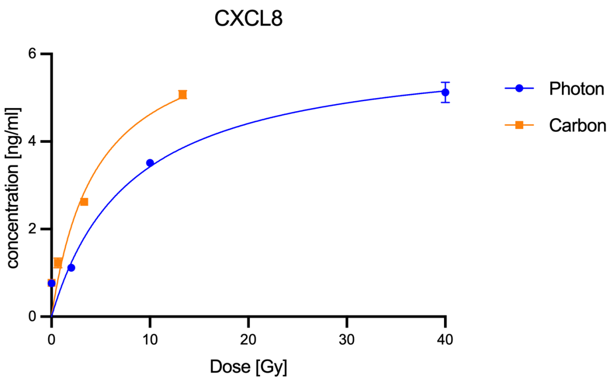

Supplemental Figure S8. Photon and particle irradiation can induce senescence with context-dependent higher biological effectiveness of carbon ions regarding SASP chemokine release. Related to Figure 5. **A,** Representative images of SA-β-Gal stained PANC-1 cancer cells irradiated with indicated doses of photons or particles. **B,** Physical equivalent doses of carbon ions induce higher release of CCL20 and CXCL8 from SK-MEL-28 cells as determined by flow cytometric bead-based immunoassays. Indicated are concentrations (mean + SD) from n=6 independent experiments. P-values were calculated with one-way ANOVA and significance testing using the Holm-Šídák method or unpaired t-tests (zig-zac line). **C,** CXCL8 release of SK-MEL-28 melanoma cells irradiated with different doses of photon or carbon ion irradiation as determined by ELISA of conditioned media at day 5 after irradiation. Indicated are concentrations (mean ± SD) from one experiment with technical duplicates.

ID	Gender	Age (years)	UICC stage	Sample site	Histology
P01	female	79	III	pancreas	pancreatic adenocarcinoma
P02	female	52	IV	liver metastasis	pancreatic adenocarcinoma
P03	male	44	0	pancreas	pancreatic intraepithelial neoplasia
P04	male	74	II	pancreas	pancreatic adenocarcinoma

Supplemental Table S2. Patient characteristics of investigated patient-derived pancreatic tumor explants. Related to Figure 2.

SASP Factor	References
CCL2 (MCP-1)	(1-8)
CCL4 (MIP-1β)	(5, 6, 9, 10)
CCL5 (RANTES)	(1, 4-7, 11-13)
CXCL1 (GRO-a)	(1, 2, 6, 7, 12, 14-17)
CXCL8 (IL-8)	(1, 2, 6, 15, 17-21)
CXCL10 (IP-10)	(2, 5, 6, 16, 22, 23)
ICAM1	(2, 6, 15, 17, 21, 24, 25)
IL1a	(1, 2, 4, 6, 7, 15-19, 26)
IL6	(1, 2, 6, 7, 12, 15-19, 21)
TNFa	(5, 6, 12, 22, 27, 28)

Supplemental Table S3. References for established SASP factors used for the SASP factor score. Related to Figure 2.

Supplemental References

1. Faget DV, Ren Q, Stewart SA. Unmasking senescence: context-dependent effects of SASP in cancer. Nature Reviews Cancer. 2019;19(8):439-53.
2. González-Gualda E, Baker AG, Fruk L, Muñoz-Espín D. A guide to assessing cellular senescence in vitro and in vivo. The FEBS journal. 2021;288(1):56-80.
3. Iannello A, Thompson TW, Ardolino M, Lowe SW, Raulet DH. p53-dependent chemokine production by senescent tumor cells supports NKG2D-dependent tumor elimination by natural killer cells. The Journal of experimental medicine. 2013;210(10):2057-69.
4. Kang TW, Yevsa T, Woller N, Hoenicke L, Wuestefeld T, Dauch D, et al. Senescence surveillance of pre-malignant hepatocytes limits liver cancer development. Nature. 2011;479(7374):547-51.
5. Ruscetti M, Leibold J, Bott MJ, Fennell M, Kulick A, Salgado NR, et al. NK cell-mediated cytotoxicity contributes to tumor control by a cytostatic drug combination. Science. 2018;362(6421):1416-22.
6. Saul D, Kosinsky RL, Atkinson EJ, Doolittle ML, Zhang X, LeBrasseur NK, et al. A new gene set identifies senescent cells and predicts senescence-associated pathways across tissues. Nature Communications. 2022;13(1):4827.
7. Wang L, Lankhorst L, Bernards R. Exploiting senescence for the treatment of cancer. Nature Reviews Cancer. 2022;22(6):340-55.
8. Xue W, Zender L, Miething C, Dickins RA, Hernando E, Krizhanovsky V, et al. Senescence and tumour clearance is triggered by p53 restoration in murine liver carcinomas. Nature. 2007;445(7128):656-60.
9. Parajuli P, Rosati R, Mamdani H, Wright RE, 3rd, Hussain Z, Naeem A, et al. Senescence-associated secretory proteins induced in lung adenocarcinoma by extended treatment with dexamethasone enhance migration and activation of lymphocytes. Cancer immunology, immunotherapy : CII. 2023;72(5):1273-84.
10. Schafer MJ, Zhang X, Kumar A, Atkinson EJ, Zhu Y, Jachim S, et al. The senescence-associated secretome as an indicator of age and medical risk. JCI Insight. 2020;5(12).
11. Jackson JG, Pant V, Li Q, Chang LL, Quintás-Cardama A, Garza D, et al. p53-mediated senescence impairs the apoptotic response to chemotherapy and clinical outcome in breast cancer. Cancer cell. 2012;21(6):793-806.
12. Ruscetti M, Morris JPt, Mezzadra R, Russell J, Leibold J, Romesser PB, et al. Senescence-Induced Vascular Remodeling Creates Therapeutic Vulnerabilities in Pancreas Cancer. Cell. 2020.
13. Vilgelm AE, Johnson CA, Prasad N, Yang J, Chen SC, Ayers GD, et al. Connecting the Dots: Therapy-Induced Senescence and a Tumor-Suppressive Immune Microenvironment. Journal of the National Cancer Institute. 2016;108(6):djv406.
14. Acosta JC, O'Loghlen A, Banito A, Guijarro MV, Augert A, Raguz S, et al. Chemokine Signaling via the CXCR2 Receptor Reinforces Senescence. Cell. 2008;133(6):1006-18.

15. Coppé JP, Desprez PY, Krtolica A, Campisi J. The senescence-associated secretory phenotype: the dark side of tumor suppression. Annual review of pathology. 2010;5:99-118.
16. Demaria M, Leary MN, Chang J, Shao L, Liu S, Alimirah F, et al. Cellular Senescence Promotes Adverse Effects of Chemotherapy and Cancer Relapse. Cancer discovery. 2017;7(2):165.
17. Gorgoulis V, Adams PD, Alimonti A, Bennett DC, Bischof O, Bishop C, et al. Cellular Senescence: Defining a Path Forward. Cell. 2019;179(4):813-27.
18. Hernandez-Segura A, Nehme J, Demaria M. Hallmarks of Cellular Senescence. Trends in Cell Biology. 2018;28(6):436-53.
19. Orjalo AV, Bhaumik D, Gengler BK, Scott GK, Campisi J. Cell surface-bound IL-1alpha is an upstream regulator of the senescence-associated IL-6/IL-8 cytokine network. Proc Natl Acad Sci U S A. 2009;106(40):17031-6.
20. Walle T, Bajaj S, Kraske JA, Rösner T, Cussigh CS, Kälber KA, et al. Cytokine release syndrome-like serum responses after COVID-19 vaccination are frequent and clinically inapparent under cancer immunotherapy. Nature Cancer. 2022;3(9):1039-51.
21. Chien Y, Scuoppo C, Wang X, Fang X, Balgley B, Bolden JE, et al. Control of the senescence-associated secretory phenotype by NF-κB promotes senescence and enhances chemosensitivity. Genes & development. 2011;25(20):2125-36.
22. Gluck S, Guey B, Gulen MF, Wolter K, Kang TW, Schmacke NA, et al. Innate immune sensing of cytosolic chromatin fragments through cGAS promotes senescence. Nature cell biology. 2017;19(9):1061-70.
23. Thompson PJ, Shah A, Ntranos V, Van Gool F, Atkinson M, Bhushan A. Targeted Elimination of Senescent Beta Cells Prevents Type 1 Diabetes. Cell Metab. 2019;29(5):1045-60.e10.
24. Coppé J-P, Patil CK, Rodier F, Sun Y, Muñoz DP, Goldstein J, et al. Senescence-associated secretory phenotypes reveal cell-nonautonomous functions of oncogenic RAS and the p53 tumor suppressor. PLoS Biol. 2008;6(12):2853-68.
25. Gorgoulis VG, Pratsinis H, Zacharatos P, Demoliou C, Sigala F, Asimacopoulos PJ, et al. p53-Dependent ICAM-1 overexpression in senescent human cells identified in atherosclerotic lesions. Laboratory Investigation. 2005;85(4):502-11.
26. Acosta JC, Banito A, Wuestefeld T, Georgilis A, Janich P, Morton JP, et al. A complex secretory program orchestrated by the inflammasome controls paracrine senescence. Nature cell biology. 2013;15(8):978-90.
27. Huang W, Hickson LJ, Eirin A, Kirkland JL, Lerman LO. Cellular senescence: the good, the bad and the unknown. Nature Reviews Nephrology. 2022;18(10):611-27.
28. Takasugi M, Yoshida Y, Hara E, Ohtani N. The role of cellular senescence and SASP in tumour microenvironment. The FEBS journal. 2023;290(5):1348-61.
