# Supplementary material for: Photon and particle radiotherapy induce redundant modular chemotaxis of human lymphocytes
Source: JCI Insight. 2025 Aug 14;10(18):e190149. doi: 10.1172/jci.insight.190149 (PMC12487834; doi:10.1172/jci.insight.190149)
Supplement: Unedited blot and gel images [file jciinsight-10-190149-s245.pdf]

p21

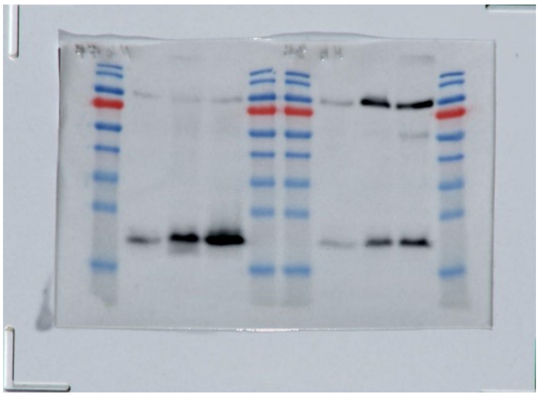

β-actin

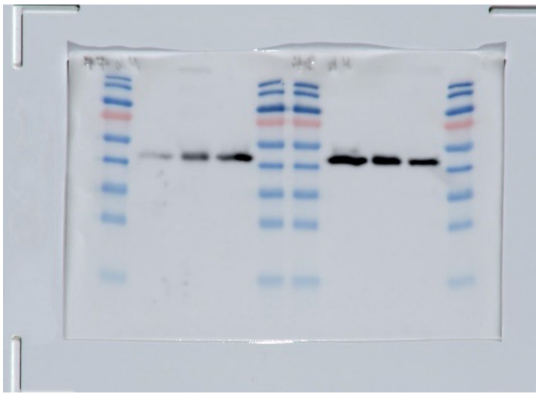

p21

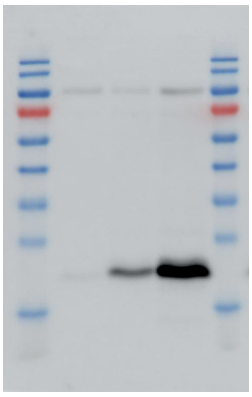

β-actin

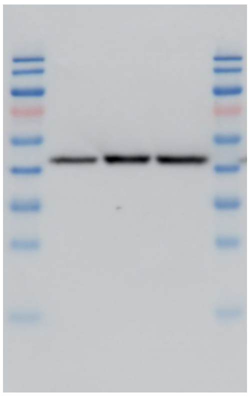

Full unedited western blot gels. Related to Figure 2B. Shown are full length original immunoblot images for p21 and β-actin expression in pancreatic tumor explants treated with 0 Gy, 10 Gy or 20 Gy. Lanes from left to right: patient #1 0Gy, patient #1 10Gy, patient #1 20Gy, patient #2 0Gy, patient #2 10Gy, patient #2 20Gy (upper panel); patient #4 0Gy, patient #4 10Gy, patient #4 20Gy (lower panel).
